# Efficient production of hydroxysalidroside in *Escherichia coli* via enhanced glycosylation and semi-rational design of *UGT85A1*

**DOI:** 10.1016/j.synbio.2025.03.002

**Published:** 2025-03-06

**Authors:** Xinru Wang, Lian Wang, Qihang Chen, Ke Wang, Huijing Wang, Dong Li, Song Gao, Weizhu Zeng, Jingwen Zhou

**Affiliations:** a Engineering Research Center of Ministry of Education on Food Synthetic Biotechnology, Jiangnan University, 1800 Lihu Road, Wuxi, Jiangsu 214122, China; b Science Center for Future Foods, Jiangnan University, 1800 Lihu Road, Wuxi, Jiangsu 214122, China; c Key Laboratory of Industrial Biotechnology, Ministry of Education and School of Biotechnology, Jiangnan University, 1800 Lihu Road, Wuxi, Jiangsu 214122, China; d Jiangsu Province Engineering Research Center of Food Synthetic Biotechnology, Jiangnan University, Wuxi 214122, China

**Keywords:** *Escherichia coli*, Hydroxysalidroside, Semi-rational design, UDPG, *UGT85A1*

## Abstract

Hydroxysalidroside is an important natural phenylethanoid glycoside with broad application prospects in the food and pharmaceutical fields. However, its low concentration in plants and complex extraction hinder its production. Despite being a promising way to synthesize hydroxysalidroside in *Escherichia coli*, glycosylation remains the limiting factor for its production. A *de novo* biosynthetic pathway for hydroxysalidroside was successfully constructed in *E. coli* via the screening of glycosyltransferase, overexpressing phosphoglucomutase (*pgm*) and UDP-glucose pyrophosphorylase (*galU*) to ensure a sufficient supply of UDP-glucose (UDPG). Additionally, a semi-rational design of *UGT85A1* was conducted to expand the acceptor-binding pocket to eliminate steric hindrance interfering with the binding of hydroxytyrosol. The endogenous genes *ushA* and *otsA* were knocked out to further reduce the consumption of UDPG. Finally, a titer of 5837.2 mg/L was achieved in a 5 L fermenter by optimizing the feeding times of carbon sources. This laid the foundation for the subsequent biosynthesis of phenylethanoid glycosides.

## 1 Introduction

Hydroxysalidroside is an important naturally occurring phenylethanoid glycoside derived from plants [1], identified as the β-D-pyranoglucoside of hydroxytyrosol [2]. The primary effects of hydroxysalidroside include antioxidant properties, anti-aging benefits, and neuroprotection. It has demonstrated broad application prospects in the fields of functional foods, cosmetics, and medicine [3,4]. Glycosylation can enhance the stability and solubility of hydroxytyrosol [5,6], making long-term preservation feasible. Hydroxysalidroside exhibits antitumor activity comparable to hydroxytyrosol, with a significantly improved safety profile [7]. Hydroxysalidroside is also regarded as a potential neuroprotective agent due to its remarkable neuroprotective effects [8]. Additionally, it serves as a crucial precursor in the synthesis of verbascoside, underscoring its extensive potential for development and application [9]. Considering the functionality of hydroxysalidroside, its synthesis has garnered widespread attention. Currently, the primary production relies on plant extraction [4], which is limited by the growth cycle and synthetic ability of plants. Microbial biosynthesis offers a sustainable and economically viable alternative for producing natural phenylethanoid glycosides [[10], [11], [12], [13], [14]].

**Fig. 1:**
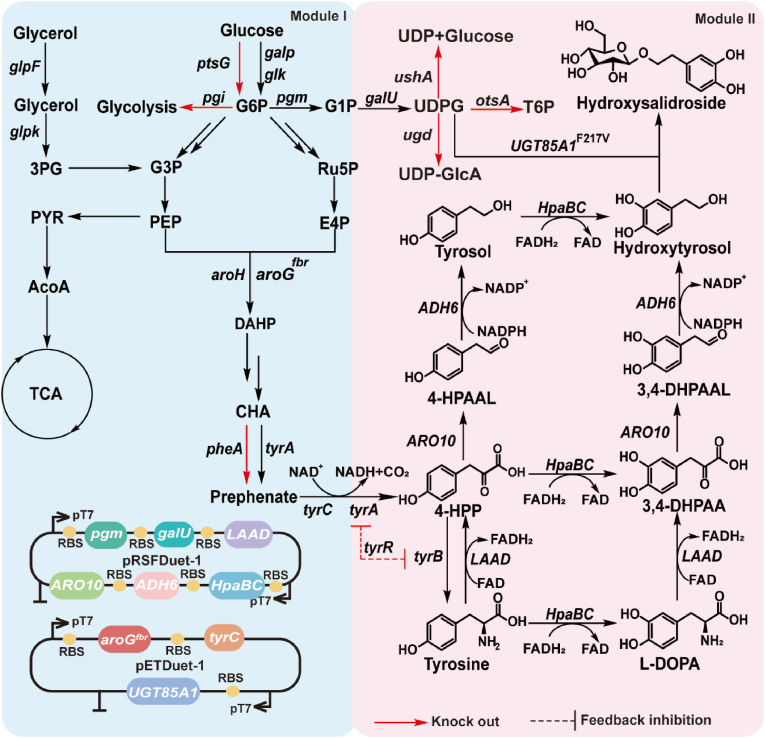
Biosynthesis of hydroxysalidroside from glucose and glycerol in *E. coli*. The blue area on the left of figure refers to the shikimate pathway. The pink area on the right of the figure refers to the synthesis module of hydroxysalidroside. *pgi*, encoding phosphoglucose isomerase; *pgm*, encoding phosphoglucomutase; *galU*, encoding glucose-1-phosphate uridylyltransferase; *ugd*, encoding UDP-glucose 6-dehydrogenase; *otsA*, encoding trehalose-6-phosphate synthase; *ushA*, encoding UDPG hydrolase; *aroG*, encoding 3-deoxy-D-arabino-heptulosonate-7-phosphate synthase; *pheA*, encoding chorismate mutase and prephenate dehydratase; *tyrA*, encoding fused chorismate mutase T/prephenate dehydrogenase; *tyrR*, aromatic amino acid biosynthesis and transport regulon transcriptional regulator; *tyrB*, encoding tyrosine aminotransferase; *tyrC*, encoding the feedback-insensitive cyclohexadienyl dehydrogenase; *ARO10*, encoding phenylpyruvate decarboxylase; *HpaB*, encoding 4-hydroxyphenylacetate 3-monooxygenase; *HpaC*, encoding 4-hydroxyphenylacetate 3-monooxygenase, reductase component; *LAAD*, encoding l-amino acid deaminase; *ADH6*, encoding alcohol dehydrogenase; and *UGT85A1*, encoding glycosyltransferase.

The biosynthesis pathway of hydroxysalidroside has two modules (Fig. 1). The first module involves the biosynthesis of 4-hydroxyphenylpyruvic (4-HPP) from simple carbon sources via the glycolysis pathway and the shikimate pathway. Subsequently, 4-HPP is converted into hydroxytyrosol through a series of deamination and hydroxylation reactions catalyzed by *ARO10*, *ADH6* (from *Saccharomyces cerevisiae*), and *HpaBC* (from *E. coli*). Finally, glycosyltransferase *UGT85A1* catalyzes the glycosylation of hydroxytyrosol, resulting in the formation of hydroxysalidroside. Previous research on hydroxysalidroside production has focused on whole-cell catalysis and mixed-strain two-step fermentation. Whole-cell catalysis is a commonly used method. *E. coli* strains were engineered to express *UGT85A1* along with the UDPG biosynthesis genes *pgm* and *galU*, achieving a titer of 6.8 g/L in a 3 L fermenter with the addition of 4 g/L hydroxytyrosol [15]. However, the high costs associated with precursor materials and the mass transfer limitations inherent to this approach present significant challenges for large-scale production. Mixed-strain two-step fermentation has also been investigated as an alternative strategy. Hydroxytyrosol was first synthesized by B-TYS6, and its filtrate was then combined with *E. coli* expressing *UGT85A1*, producing 134.2 mg/L of hydroxysalidroside [16]. The complexity of monitoring and maintaining multiple microbial strains, presents significant obstacles to industrial application. To address these limitations, constructing a *de novo* biosynthetic pathway while enhancing glycosylation efficiency provides a promising strategy, enabling scalable and cost-effective hydroxysalidroside production for industrial applications.

The modification of plant glycosyltransferases through protein engineering is an effective strategy for increasing catalytic efficiency and further enhancing the efficiency of glycosylation reactions during the synthesis of glycoside products. For instance, *UGT74AC1* has been engineered based on its crystal structure, resulting in several mutants demonstrating enhanced catalytic activities by directed evolution [17]. However, engineering glycosyltransferases without structures is associated with the following challenges: (1) uncertainty due to the lack of structural and functional information and (2) substantial screening burden resulting from the large number of mutants [18]. Semi-rational design tackles these challenges by utilizing sequence analysis to reduce uncertainty and applying targeted mutagenesis to minimize the screening burden while enhancing enzyme efficiency [19]. Saturation mutagenesis was performed on key residues within the substrate pocket of *UGT*_*BL*_*1* and *UGT51* using a semi-rational design approach, enhancing regioselectivity and catalytic activity [20,21]. However, this strategy still faces the challenge of establishing efficient screening methods, making further reduction of mutant numbers the key to addressing this issue. The availability of UDPG is another key factor to determine glycosylation reactions. Recent studies have established a UDPG regeneration system using a combination of glycosyltransferase and sucrose synthase to improve the effective supply of intracellular UDPG [22,23]. Furthermore, the overexpression of genes related to UDPG synthesis is essential for increasing the availability of UDPG [24,25]. Beyond improving UDPG supply, another effective strategy is to reduce its consumption by blocking competing pathways, thereby directing more UDPG toward the glycosylation of the target product [26]. However, how to maximize the use of UDPG remains an issue during the biosynthesis of hydroxysalidroside.

In this study, we developed a *de novo* synthesis pathway for efficiently producing hydroxysalidroside in *E. coli* via chassis engineering and semi-rational design. Chassis engineering was employed to enhance UDPG availability, addressing the issue of inadequate UDPG supply. The catalytic activity of *UGT85A1* was enhanced through semi-rational design using a focused, rational iterative site-specific mutagenesis (FRISM) [27] protocol to minimize the number of mutations. The catalytic activity of mutant *UGT85A1*^F217V^ increased compared with that of the wild type. The final titer was 5837.2 mg/L, the highest reported for biosynthesis to date. This study also established a foundation for the production of phenylethanoid glycosides.

## 2 Materials and methods

### 2.1 Strains and chemicals

*E. coli JM109* was used for plasmid construction and preservation. *E. coli* BL21(DE3) was used for the production of hydroxysalidroside. All strains constructed in this study are listed in Table S1. Luria-Bertani (LB) medium (10 g/L tryptone, 5 g/L yeast extract, and 10 g/L NaCl) was used for seed culture. The fermentation medium [28] (10 g/L glycerol, 25 g/L glucose, 7 g/L yeast extract, 7.5 g/L (NH_4_)_2_SO_4_, 3 g/L K_2_HPO_4_·3H_2_O, 2 g/L KH_2_PO_4_, 1 g/L MgSO_4_·7H_2_O, 1.1 g/L citric acid monohydrate, 0.1 g/L thiamine·HCl, 0.45 g/L ascorbic acid, and 1 mL/L trace element solution) was used for shake-flask and scale-up fermentation to accumulate the production of hydroxysalidroside. The Terrific Broth (TB) medium (5 g/L glycerol, 12 g/L tryptone, 24 g/L yeast extract, 16.37 g/L K_2_HPO_4_·3H_2_O, and 2.31 g/L KH_2_PO_4_) was used for the cultivation of protein-expressing strains. The yeast extract and tryptone were purchased from Angel (Hubei, China), whereas other chemicals were purchased from Sangon (Shanghai, China). Standards were purchased from Chemfaces (Wuhan, China) and Aladdin (Shanghai, China).

### 2.2 Plasmid construction

All plasmids constructed and primers used in this study are listed in Table S2 and Table S3, respectively. To construct pETDuet-*UGT85A1*, pETDuet-*UGT33*, and pETDuet-*UGT13*, UDPG-dependent glycosyltransferase from *Arabidopsis thaliana* (*UGT85A1*, accession number: NP_173656.1), *Rhodiola rosea* (accession number: MF674558.1), and *Oryza sativa* (accession number: XP_015622802.1) were synthesized by Sangon. pETDuet-1 was linearized using Duet-F and Duet-R. The genes encoding 4-hydroxyphenylacetate 3-hydroxylase complex (*HpaBC*), phosphoglucomutase (*pgm*), and UDP-glucose pyrophosphorylase (*galU*) were amplified separately from *E. coli* BL21(DE3) genomic DNA using the primers *HpaBC*-F/*HpaBC*-R, *pgm*-F/*pgm*-R, and *galU*-F/*galU*-R. The pETDuet-*aroG*^fbr^-*tyrC* [29], which was previously constructed, was linearized with CDF-M2-F and CDF-M2-R and then assembled with *UGT85A1* using a seamless assembly kit from ABclonal Technology (Wuhan, China) to construct pETDuet-*aroG*^fbr^-*tyrC*-*UGT85A1*. Similarly, pETDuet-*aroG*^fbr^-*tyrC*-*UGT85A1*-*HpaBC* and pETDuet-*aroG*^fbr^-*tyrC*-*HpaBC*-*UGT85A1*, pRSFDuet-*pgm*-*galU*-*LAAD*-*ARO10*-*ADH6*, pRSFDuet-*pgm*-*galU*-*LAAD*-*HpaBC*-*ARO10*-*ADH6*, and pRSFDuet-*pgm*-*galU*-*LAAD*-*ARO10*-*ADH6*-*HpaBC* were constructed. *ARO10* (accession number: EF059264.1) and *ADH6* (accession number: NP_014051.3) were amplified from *Saccharomyces cerevisiae* genomic DNA using primers *ARO1*0-F/R and *ADH6*-F/R. The primers *pgm*-F and *galU*-R, *ARO10*-F, and *ADH6*-R were used for obtaining *pgm*-*galU* and *ARO10*-*ADH6* by fusion PCR. Then, the synthesized fragment *LAAD* from *Proteus mirabilis* (accession number: AXQ04983.1) was used to obtain *pgm*-*galU*-*LAAD*. All *UGT85A1* mutants were generated using the pETDuet-*aroG*^fbr^-*tyrC*-*UGT85A1* plasmid as a template, with primers designed to target the mutation site for PCR amplification. pET28a-1 was linearized using pET28a-F and pET28a-R, then separately assembled with *UGT85A1* and *UGT85A1*^F217V^. Following PCR, the template plasmid was eliminated using *Dpn*I, and the fragments were assembled using the Gibson assembly method.

### 2.3 Genome modification

*ushA*, *otsA*, *ugd*, and *pgi* were knocked out using the CRISPR-Cas9 two-plasmid system [30]. The sequence of N20 for each pTargetF plasmid was predicted using the Cas-Designer (http://www.rgenome.net/cas-designer/). Related primer sequences are listed in Table S3. The corresponding primers were used to construct upstream and downstream homologous fragments, and then fusion PCR was used to connect upstream and downstream fragments as knockout homologous fragments. The *E. coli* BL21(DE3) Δ*tyrR*Δ*ptsG*Δ*crr*Δ*pheA* [31,32] was transformed with pCas9 plasmid, homologous fragments, and pTargetF plasmid. The transformants were spread onto agar plates containing streptomycin (50 μg/mL) and kanamycin (50 μg/mL). Positive strains were confirmed by colony PCR and DNA sequencing. The pTargetF plasmid was removed by inducing with 10 mM rhamnose, while the pCas9 plasmid was eliminated through the addition of 10 mM sucrose.

### 2.4 Fermentation conditions

Shake-flask fermentation was accomplished in 250 mL Erlenmeyer flasks containing 25 mL of fermentation medium. Each Erlenmeyer flask was filled with 0.3 g CaCO_3_ before sterilization. To ensure plasmid stability during fermentation, an antibiotic mixture (ampicillin: 100 mg/mL, kanamycin: 50 mg/mL) was added at a final concentration of 0.1 % (*v*/*v*) to both LB and fermentation medium. The *E. coli* colonies were inoculated into 4 mL of LB medium and then cultured at 37 °C and 220 rpm for 8–10 h. The seed culture was inoculated into the shake flask at 2 % (*v*/*v*) of the fermentation medium volume. When the OD_600_ reached 0.8–1.0, IPTG (0.1 mM) was added and the temperature was changed to 30 °C.

A 5 L fermenter with 2.5 L of the medium with 0.1 % (*v*/*v*) antibiotic mixture was used for fed-batch fermentation. The strains were streaked on agar plates, cultured overnight, then inoculated into LB with corresponding antibiotics. After 8–10 h of fermentation, the first seed liquid was inoculated into the fermentation medium at a ratio of 4 % (*v*/*v*). The second seed liquid was inoculated into a 5 L bioreactor at a proportion of 6 % (*v*/*v*). The air solution was set at 2.5 L/min, and the temperature and pH were separately controlled at 37 °C ± 0.1 °C and 6.7 ± 0.1. Further, 4 M NaOH was used for controlling the pH. Agitation was maintained between 300 and 800 rpm, and the dissolved oxygen was set at 30 %. When OD_600_ reached approximately 30, the temperature was reduced to 30 °C, and 0.1 mM IPTG was added to the fermenter. Further, two supplementary culture mediums (Solution A, 600 g/L glycerol, 0.45 g/L ascorbic acid, and 10 g/L (NH_4_)_2_SO_4_; Solution B, 400 g/L glucose) were prepared to ensure the concentrations of glucose and glycerol within 10 g/L.

### 2.5 Molecular docking and molecular dynamics simulations

The structures of *UGT85A1* and *UGT85A1*^F217V^ were predicted using AlphaFold2. The 3D conformers of ligands UDPG and hydroxytyrosol were obtained from PubChem (https://pubchem.ncbi.nlm.nih.gov/). The docking models were constructed using Discovery Studio 2019. The preparation of the receptor *UGT85A1* and ligands included removing water and adding hydrogen and atomic charges. CDOCKER protocol was used to minimize and dock the prepared ligands into the protein receptor. PyMol 2.5.2 was used for visualizing docking results. Methods from previous studies were used as references to perform molecular dynamics simulations [33].

### 2.6 Purification and kinetic parameters determination of *UGT85A1* and *UGT85A1*^F217V^

pET28a-*UGT85A1* and pET28a-*UGT85A1*^F217V^ were separately transformed into *E*. *coli* BL21(DE3). The strains with corresponding plasmids were cultured in the TB medium with kanamycin (50 μg/mL) at 37 °C. When the OD_600_ reached 0.6–0.8, protein expression was induced by 0.1 mM IPTG was at 20 °C for 20 h. Protein purification was performed with reference to previous studies [34].

The kinetic parameters were measured in 500 μL reaction mixtures with 10 mM UDPG, 50 mM Tris-HCl (pH 7.0), a certain amount of enzymes (1–10 μg), and different concentrations of hydroxytyrosol (0.05–5 mM) at 30 °C for 30 min. Then, 500 μL of acetonitrile was added to terminate the reaction. The mixture was then clarified by centrifugation at 14,000 *g* for 10 min. The supernatant was immediately analyzed by HPLC.

### 2.7 Analytical methods

After violently shaking, the samples were diluted with methanol as needed and centrifuged at 14,000 *g* for 10 min. The supernatant was filtered through a 0.22 μm organic filter membrane and analyzed using HPLC (Shimadzu LC-20A system, Tokyo, Japan) with a C18 column (4.6 × 250 mm^2^, 5 μm; Thermo Fisher Scientific, MA, USA) at 274 nm. The column oven temperature was 40 °C, and the flow rate was 1 mL/min. The mobile phases used during the testing process were buffers A and B. Buffer A was water with 0.1 % trifluoroacetic acid, and buffer B was acetonitrile with 0.1 % trifluoroacetic acid. The gradient elution was set as follows: 0–5 min, 2 %–10 % buffer B; 5–10 min, 10 %–60 % buffer B; 10–12 min, 60 %–90 % buffer B; 12–14 min, 90 %–2 % buffer B; 14–16 min, 2 % buffer B; and 16–20 min, 0 % buffer B. The injection volume of each sample was 10 μL. Glycerol and glucose were quantified using a refractive index detector (Shimadzu) with an Aminex HPX87H HPLC column at 40 °C. The mobile phase was 5 mM diluted sulfuric acid, and the flow rate was 0.6 mL/min.

Waters MALDI SYNAPT Q-TOF MS platform (MA, USA) equipped with a BEH C18 column (2.1 × 150 mm^2^, 1.7 μm) was used to analyze and further confirm the product structure in the fermentation process. The settings were as follows: ESI mode; capillary voltage, 3.0 kV; cone voltage, 20/50 V; source block temperature, 100 °C; desolvation temperature, 400 °C; flow of desolvation gas, 700 lit/h; flow of cone gas, 50 lit/h; collision energy, 6 eV; range of mass, 50–1000 *m*/*z*; and detector, 1800 V. Purified fermentation samples and the hydroxysalidroside standard were dissolved in acetonitrile to achieve a final concentration of 0.1 mg/mL.

## 3 Results

### 3.1 Screening and validation of UGTs

**Fig. 2:**
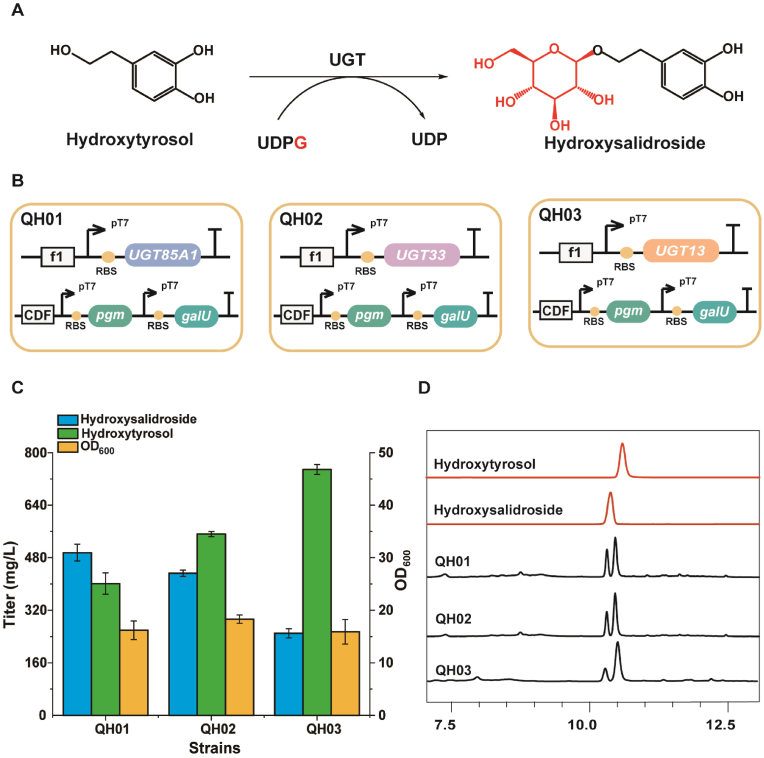
Whole-cell catalysis for producing hydroxysalidroside from hydroxytyrosol. (A) Glycosylation of hydroxytyrosol via UGT. (B) Construction of a strain for salidroside production via dual-plasmid fermentation. (C) Hydroxysalidroside production, hydroxytyrosol residue and OD_600_ of QH01–QH03 in the shake flask in 48 h. (D) HPLC of QH01–QH03. Data shown are mean ± SD of three independent experiments.

Hydroxysalidroside was synthesized from UDPG and hydroxytyrosol (Fig. 2A). UGTs with regio-specific T8GT activity from different plant species were selected and expressed, including *UGT85A1*, *UGT33*, and *UGT13*, to identify the UDP-glycosyltransferase responsible for glycosylation. These enzymes were ligated into the plasmid pETDuet-1 and subsequently transferred into *E. coli* BL21(DE3) along with pCDFDuet-*pgm*-*galU* (Fig. 2B) to ensure a sufficient supply of UDPG, resulting in strains QH01, QH02, and QH03. The titer and structure of hydroxysalidroside were analyzed using HPLC and LC-MS (Fig. 2C and D and Fig. S1). Strain QH01 produced 495.4 mg/L hydroxysalidroside, exhibiting the highest substrate conversion among the three strains. The product had a molecular ion at *m*/*z* 315.1093 ([M − H]^–^), matching that of the hydroxysalidroside standard. Therefore, *UGT85A1* was chosen for biosynthesizing hydroxysalidroside.

### 3.2 Construction of the hydroxysalidroside synthetic pathway

**Fig. 3:**
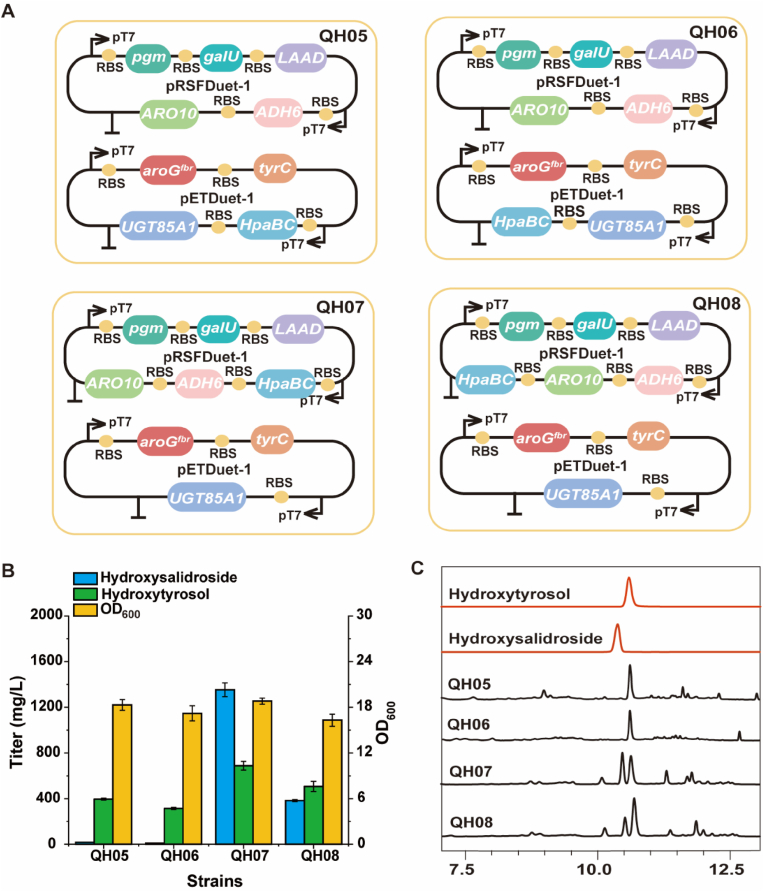
Effect of hydroxylation efficiency on the synthesis of hydroxysalidroside. (A) Hydroxylation efficiency was changed by tuning the expression level of *HpaBC*. (B) Hydroxysalidroside synthesis analysis in 48 h. (C) HPLC detection of QH05–08. Data shown are mean ± SD of three independent experiments.

A sufficient supply of the precursor hydroxytyrosol was ensured by the hydroxylation efficiency. To construct the *de novo* hydroxysalidroside biosynthetic strains, the previously constructed strain *E. coli* BL21(DE3) Δ*tyrR*Δ*ptsG*Δ*crr*Δ*pheA* [31,32] (strain QH04) was chosen as the chassis. *HpaBC* (encoding 4-hydroxyphenylacetate 3-hydroxylase complex), *ADH6* (encoding alcohol dehydrogenase), and *ARO10* (encoding phenylpyruvate decarboxylase) were overexpressed to produce hydroxytyrosol. *LAAD* (encoding l-amino acid deaminase) was used for promoting the regeneration of FADH_2_ and increasing the metabolic flux. *UGT85A1* was inserted into the previously constructed plasmid pETDuet-*aroG*^fbr^-*tyrC* (*aroG*^fbr^, encoding 3-deoxy-D-arabino-heptulosonate-7-phosphate synthase; *tyrC*, encoding the feedback-insensitive cyclohexadienyl dehydrogenase), which was designed to alleviate tyrosine feedback inhibition to achieve glycosylation (Fig. 3A) [29]. The hydroxylation reaction catalyzed by *HpaBC* is crucial for synthesizing hydroxytyrosol. The effect of different expression vectors and connection patterns for *HpaBC* was evaluated using strains QH05–QH08. The titer of hydroxysalidroside after 48 h was 17.2, 9.3, 1353.8, and 382.3 mg/L for QH05, QH06, QH07, and QH08; OD_600_ was 18.3, 17.2, 18.8, and 16.3, respectively (Fig. 3B and C). The residual hydroxytyrosol in QH07 was 687.3 mg/L. Overall, QH07 exhibited the highest hydroxylation efficiency.

Among strains QH05–QH08, *HpaBC* genes were inserted into different plasmids (pETDuet-1 and pRSFDuet-1) at different positions (Fig. 3A). Therefore, their expression levels were influenced not only by plasmid copy number [35] but also by the distance from the promoter [36]. The *HpaBC* is encoded by the *HpaB* (4-hydroxyphenylacetate 3-monooxygenase) and *HpaC* (4-hydroxyphenylacetate 3-monooxygenase, reductase component) genes [37]. *HpaB* (58.8 kDa), which is primarily responsible for the hydroxylation process, was selected as the target protein for analysis. SDS-PAGE was used to verify the expression levels of *HpaB* across different strains. The results (Fig. S2) demonstrate variations in expression levels among the strains, with QH07 exhibiting the highest *HpaB* expression, consistent with the fermentation results.

### 3.3 Increasing UDPG flux enhanced glycosylation by chassis engineering

**Fig. 4:**
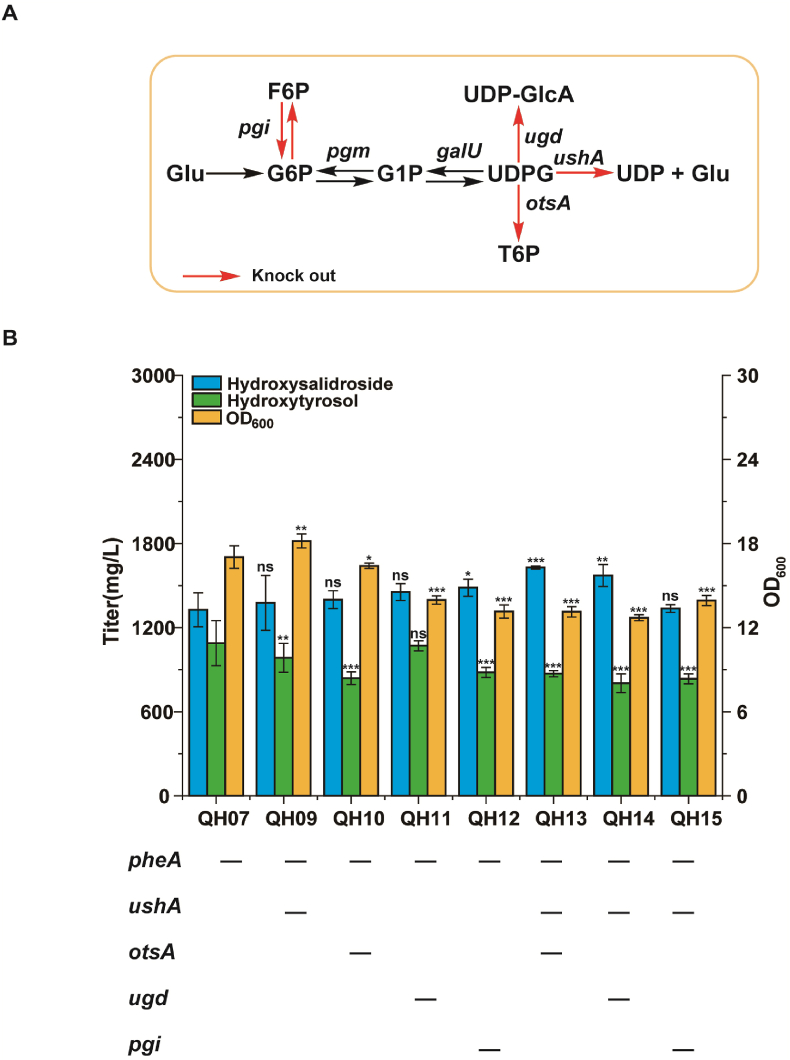
Improving the titer of hydroxysalidroside through enhanced glycosylation in *E. coli*. (A) Engineering the UDP-glucose biosynthesis pathway in *E. coli*. (B) Product accumulation in QH07 and QH09–QH15 with modified UDP-glucose biosynthesis pathway. Data shown are mean ± SD of three independent experiments. Statistical significance was determined using one-way ANOVA followed by Tukey's post-hoc test. Asterisks indicate significant differences (∗p ≤ 0.05, ∗∗p ≤ 0.01, ∗∗∗p ≤ 0.001).

Several metabolic pathways consume UDPG, significantly limiting the availability of UDPG in *E. coli*. UDPG hydrolase (*ushA*), alglucose-6-phosphate synthase (*otsA*), and UDP-glucose 6-dehydrogenase (*ugd*) can significantly hinder the biosynthesis and accumulation of UDPG (Fig. 4A). Consequently, these genes were initially knocked out to generate strains QH09–QH11, with titers increasing to 1376.2, 1398.8, and 1451.1 mg/L in 48 h, respectively (Fig. 4B). Furthermore, knocking out the key gene *pgi* of the Embden-Meyerhof-Parnas (EMP) pathway increased the titer to 1484.7 mg/L (strain QH12). Based on strain QH09, additional gene knockouts were performed (strains QH13–15), and it was evident that QH13 achieved the highest titer of 1628.9 mg/L. Although the metabolic modification of endogenous UDPG improved glycosylation efficiency, hydroxytyrosol still partially accumulated.

### 3.4 Semi-rational design of *UGT85A1*

**Fig. 5:**
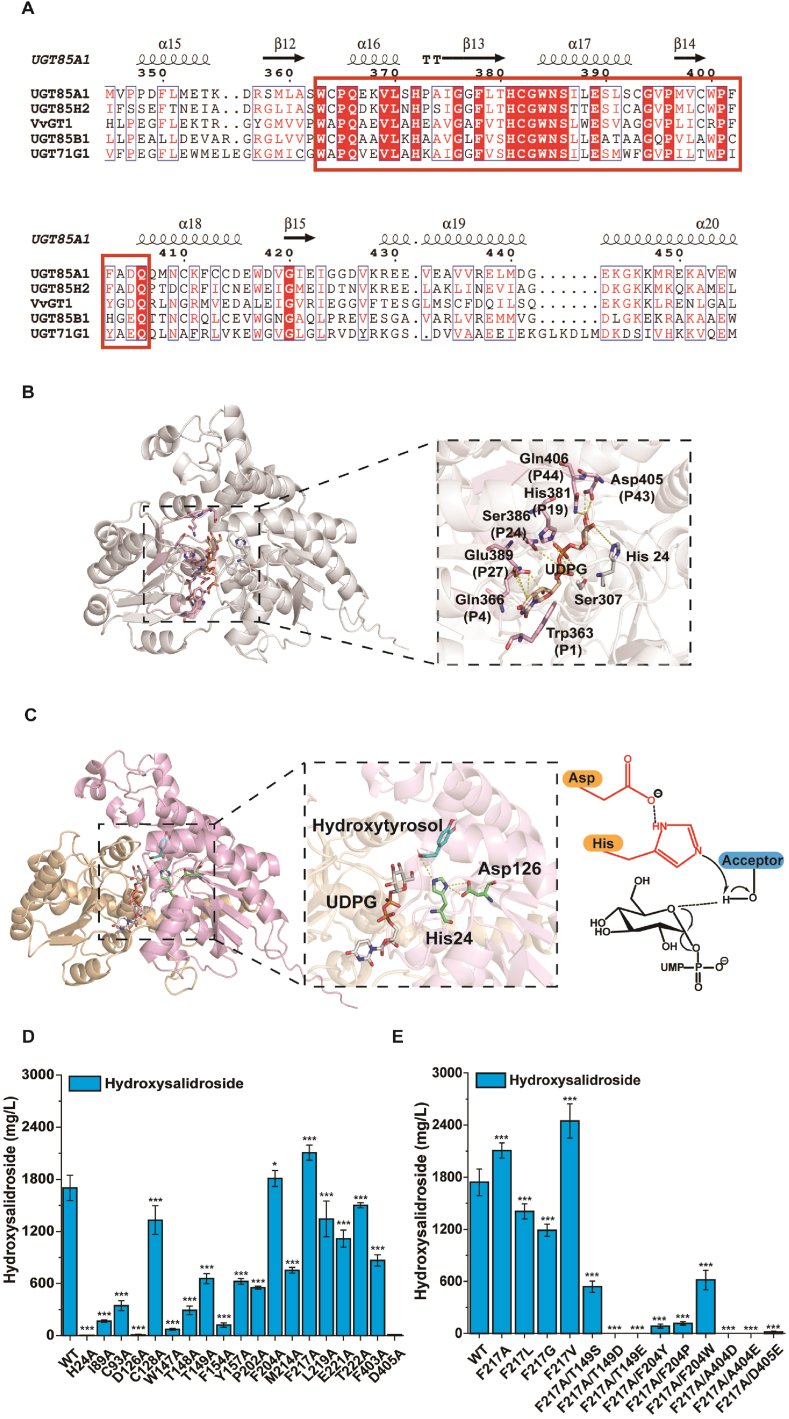
Enhancing the catalytic efficiency of *UGT85A1* through protein engineering. (A) Multiple sequence alignment with known crystal structures of UGTs. (B) Identification of the UDPG-binding pocket based on the PSPG region. (C) Catalytic mechanism of *UGT85A1*. (D) Shake-flask fermentation results after alanine mutation. (E) Semi-rational design of mutation sites and verification through shake-flask fermentation. Data shown are mean ± SD of three independent experiments. Statistical significance was determined using one-way ANOVA followed by Tukey's post-hoc test. Asterisks indicate significant differences (∗p ≤ 0.05, ∗∗p ≤ 0.01, ∗∗∗p ≤ 0.001).

Besides increasing the supply of endogenous UDPG, the glycosylation efficiency was further improved by modifying *UGT85A1*. Multiple sequence alignment was used to align the amino acid sequence of *UGT85A1* and other UGTs with resolved crystal structures (Fig. 5A). Therefore, the highly conserved PSPG region (from W363 to Q406) in the C-terminal domain (CTD) of the enzyme was identified. PSPG region is essential for defining the key residues of the UDPG-binding pocket [19]. Hence, the docking of the glycosyl donor UDPG was completed (Fig. 5B). Previous reports confirmed that the acceptor-binding pocket is located in the N-terminal domain (NTD) of UGTs [19]. Subsequently, the substrate hydroxytyrosol was docked into the *UGT85A1* structure (Fig. 5C). Various successful attempts to engineer UGTs through structure-guided design highlighted the significance of amino acid residues near the acceptor-binding pockets in influencing the activity [19]. Hence, the residues surrounding the binding site for hydroxytyrosol were evaluated for possible mutagenesis.

After the virtual alanine scan, 19 residues within a 0.5-nm range of the substrate were identified as hotspots that could significantly impact the catalytic activity of *UGT85A1*. All these mutations were constructed and subsequently introduced into strain QH13, and the catalytic activity was analyzed after 48 h of shake-flask fermentation. The results indicated that only five strains with mutants achieved a production titer reaching at least 75 % of that achieved by QH13 expressing wild-type *UGT85A1* (WT). Among these, the titer of strains F204A and F217A reached 1810.7 and 2107.1 mg/L, and the titer increased by 6.3 % and 23.8 %, respectively. Further, the strains H24A, D126A, and D405A were almost inactive (Fig. 5D). H24 and D126 were confirmed to be the highly conserved catalytic residues. They are responsible for deprotonating the hydroxyl group of the acceptor molecule and balancing the charge, respectively [17,38] (Fig. 5C). D405 is the penultimate amino acid residue in the PSPG motif, which directly interacts with the glycosyl donor [38]. Therefore, the mutation of D405 to alanine significantly impaired the catalytic activity of the enzyme.

The second round of mutagenesis on *UGT85A1* was conducted using the FRISM method. F217A was the best variant found in the first round of evolution, and it was chosen as the template for the second round. It was speculated that the decrease in steric hindrance caused by the reduction of side chains made it easier for substrates to enter the catalytic pocket, resulting in an increased titer. Strains F217L, F217G, and F217V were constructed to test this hypothesis. The titer of strain F204A also increased compared with that of WT. Considering the cyclic structure in the amino acid side chain of F204, an attempt was made to generate the mutant strains F217A/F204Y, F217A/F204P, and F217A/F204W. The docking result showed that T149, A404, and D405 were all located near the alcohol hydroxyl group of hydroxytyrosol in the spatial position. The location indicated that these were the potential amino acid groups affecting glycosylation reactions. Therefore, electron-rich amino acids with more similar structural properties (S, D, and E) were introduced into these sites. After constructing and screening, the best mutant strain identified was F217V (strain QH16), which showed a 50.2 % improvement compared with the WT, with the titer reaching 2447.3 mg/L (Fig. 5E).

The kinetic parameters of *UGT85A1*^F217V^ were determined in comparison with *UGT85A1* (Table S5). The *K*_m_ of *UGT85A1*^F217V^ was 0.47 ± 0.04 mM, which was lower than *UGT85A1*, indicating an enhanced substrate affinity. Meanwhile, the *k*_cat_ value of *UGT85A1*^F217V^ increased 42.5 % compared to *UGT85A1*, suggesting a remarkable improvement in catalytic efficiency. These results showed that the improved catalytic performance of *UGT85A1*^F217V^ contributed to the increased production of hydroxysalidroside. We also measured OD_600_ and hydroxytyrosol titer (Fig. S3) to better demonstrate that the increase in production was due to the enhanced activity of the *UGT85A1*^F217V^.

### 3.5 Analysis of the mechanism of *UGT85A1*^F217V^catalytic efficiency improvement

**Fig. 6:**
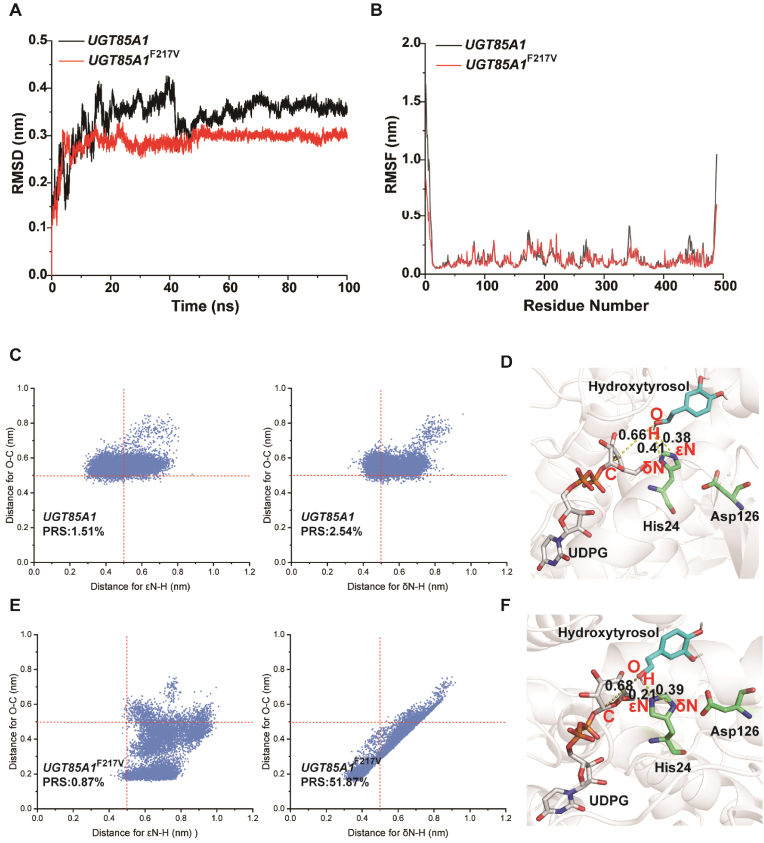
**Results of molecular dynamics (MD) simulations.** (A) RMSD for *UGT85A1* and *UGT85A1*^F217V^. (B) RMSF for *UGT85A1* and *UGT85A1*^F217V^. (C) Distributions of two key distances d_εN-H_, d_δN-H_, and d_O-C_ of *UGT85A1* with PRS occupancy. The PRS numbers indicate the fraction of enzyme conformations that are considered PRSs, meaning those conformations where both key distances (d_εN-H_, d_δN-H_ and d_O-C_) are less than 0.5 nm, which are favorable for product formation. (D) Representative structure of *UGT85A1* obtained from MD simulations in 100 ns. The numbers represent the key distances (d_εN-H_, d_δN-H_, and d_O-C_) measured at 100 ns. (E) Distributions of two key distances d_εN-H_, d_δN-H_, and d_O-C_ of *UGT85A1*^F217V^ with PRS occupancy. (F) Representative structure of *UGT85A1*^F217V^ obtained from MD simulations in 100 ns.

The catalytic activity of the *UGT85A1*^F217V^ mutant was significantly enhanced compared with the *UGT85A1*. However, the molecular mechanism underlying the increase in catalytic efficiency remains unclear. The analysis of the root mean square deviation (RMSD, Fig. 6A) indicated that the structures of *UGT85A1* and *UGT85A1*^F217V^ reached equilibrium after 50 ns, confirming the overall stability of the system. Subsequently, the data were processed to obtain the root mean square fluctuation (RMSF, Fig. 6B) to characterize the range of fluctuations in amino acids. Compared with *UGT85A1*, the *UGT85A1*^F217V^ structure exhibited increased flexibility in the 200–230 region, including the mutation site. This suggested that the mutation enhanced structural flexibility, increasing the mobility of the substrate pocket, thereby facilitating substrate-enzyme binding.

According to the catalytic mechanism of UGTs, the nucleophilic attack by H24 during catalysis occurs on the hydroxyl group of hydroxytyrosol (the nucleophilic attack can be initiated by either εN or δN due to the existence of tautomers in the imidazole ring of histidine), resulting in proton abstraction. The deprotonated hydroxytyrosol then nucleophilically attacks the C1 of glucose in UDPG, forming a glycosidic bond to produce salidroside. Therefore, the distances between εN and δN of H24 to the hydroxyl hydrogen (d_εN-H_ and d_δN-H_), as well as the distance between the hydroxyl oxygen and the glucose C1 (d_O-C_), were analyzed (Fig. 6D and F). Conformations where both distances were less than 0.5 nm were considered favorable for product formation and referred to as pre-reaction states (PRSs) [39]. A comparison revealed that the PRS of d_δN-H_ and d_O-C_ in the F217V increased to 51.87 % compared with the *UGT85A1*, whereas the PRS of d_εN-H_ and d_O-C_ decreased to 0.87 % (Fig. 6C and E). Thus, the enhanced nucleophilic attack initiated by δN is a key factor contributing to the increased catalytic activity.

### 3.6 Fed-batch fermentation

**Fig. 7:**
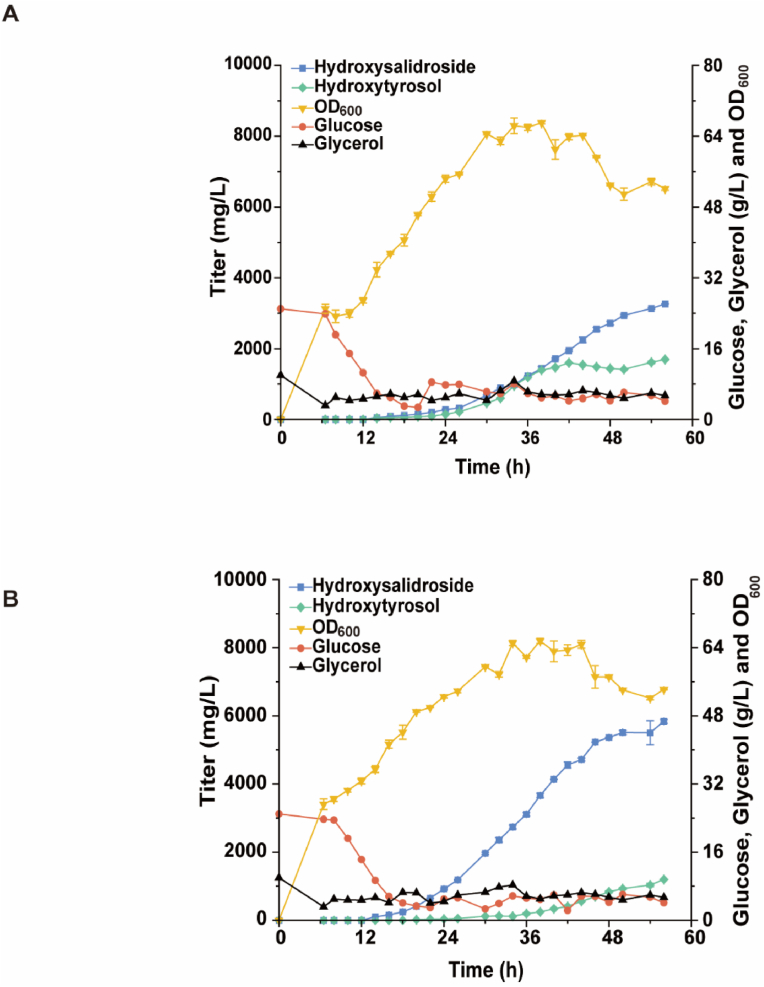
*De novo* biosynthesis of hydroxysalidroside in a 5 L fermenter. (A) Biosynthesis of hydroxysalidroside by strain QH13. (B) Biosynthesis of hydroxysalidroside by strain QH16. Data shown are mean ± SD of two independent experiments.

The strains QH13 and QH16 were cultivated in a 5 L bioreactor to evaluate the capability for hydroxysalidroside synthesis. The pH during fermentation was controlled at 6.7 to avoid the excessive oxidation of intermediate products. The pH rise was used as a signal to start feeding in the initial fermentation stage, which resulted in slow early growth of the strains. After induction, the highest OD_600_ of QH13 and QH16 only reached 56.15 and 58.3, respectively. Moreover, the production was significantly affected, and the titer of hydroxysalidroside only reached 2485.7 and 3993.8 mg/L, respectively (Fig. S3). The feeding strategy was optimized to further increase the cell density. When the real-time concentration of glycerol and glucose in the fermentation broth dropped below 5.0 g/L, the feed solutions were added to maintain their concentrations below 10.0 g/L. After adjusting the feeding method, both strains showed a significant increase in OD_600_, with maximum values reaching 67.1 and 64.8, respectively. The production also significantly increased. The titer of hydroxysalidroside in strain QH13 was 2720.7 mg/L in 48 h and further increased to 3264.1 mg/L in 56 h (Fig. 7A), which showed a 31.3 % improvement. Meanwhile, the hydroxysalidroside production of strain QH16 increased by 46.2 %, and the titer was 5837.2 mg/L in 56 h (Fig. 7B), thus achieving the current highest titer.

## 4 Discussion

Hydroxysalidroside, an important phenylethanoid glycoside, has garnered significant attention due to its antioxidant, anti-aging, and neuroprotective properties. However, challenges persist in the microbial production of hydroxysalidroside, with insufficient glycosylation efficiency being the most significant. In this study, a *de novo* biosynthetic pathway for hydroxysalidroside was constructed in *E. coli* via chassis engineering and protein engineering. The *de novo* synthesis was implemented by screening UGTs and enhancing the hydroxylation rate. The supply of UDPG was increased by enhancing its endogenous synthesis and blocking competing pathways. The semi-rational design with FRISM protocol was used to reduce screening pressure and obtain effective mutants of *UGT85A1*. A final hydroxysalidroside yield of 5837.2 mg/L was achieved in a 5 L fermenter. The biosynthesis from simple carbon sources to hydroxysalidroside was completed, laying the foundation for synthesizing other phenylethanoid glycosides.

The biosynthesis of hydroxysalidroside is affected by the glycosylation rate in *E. coli*. Hydroxysalidroside is the β-D-pyranoglucoside of hydroxytyrosol, yet native UGTs involved in its biosynthesis remain largely unexplored, making it necessary to screen alternative UGTs. Previous studies have shown that many plant UGTs exhibit substrate flexibility [[40], [41], [42]]. Enzymes such as *UGT85A1*, *UGT33*, *UGT13*, and *UGT73C5* have been confirmed to catalyze the glycosylation of tyrosol, leading to the production of salidroside [25,43,44]. Three UGTs were selected as candidates for whole-cell catalysis using hydroxytyrosol as the substrate, constructed under conditions ensuring sufficient intracellular UDPG supply. The results demonstrated that all these enzymes could catalyze the synthesis of hydroxysalidroside, with *UGT85A1* exhibiting the highest catalytic efficiency. Introducing *UGT85A1* into the synthetic pathway enabled the *de novo* synthesis of hydroxysalidroside.

The accumulation of a large amount of hydroxytyrosol indicates insufficient glycosylation efficiency in *E. coli*, highlighting the need to enhance the supply of endogenous UDPG [45,46]. *E. coli* can synthesize only a limited amount of UDPG [26]. Even with the overexpression of endogenous synthetic enzyme genes *pgm* and *galU*, further increases in the available UDPG are necessary to boost the yield of glycosylation products. Deleting *ushA* has been confirmed to be beneficial for the accumulation of glycoside products [47]. Knocking out *ugd* and *otsA* disrupts the synthesis of UDP-glucuronic acid (UDP-GlcA) and trehalose (T6P), effectively reducing UDPG consumption [48,49]. Phosphoglucose isomerase is the key enzyme in the EMP pathway encoded by *pgi*; knocking out *pgi* can significantly boost the synthesis of glycoside products, albeit with some potential impact on growth [49,50]. A PTS-deficient strain was used in this study, and glycerol and glucose were added to the medium as dual carbon sources. This approach not only promotes the synthesis of the precursor tyrosine but also allows the use of more glucose in glycosylation. Additionally, the degradation and consumption of UDPG were effectively reduced by knocking out genes such as *ushA*, *pgi*, *otsA*, and *ugd*.

Besides enhancing endogenous UDPG supply, *UGT85A1* was also engineered through semi-rational design to further improve glycosylation efficiency. Recent analyses of the crystal structures of UGTs have revealed structural similarities [51], which are of great significance for dissecting glycosylation mechanisms and guiding protein engineering modifications [19]. UGTs exhibit a GT-B folded crystal structure, composed of two Rossmann-like β/α/β domains, referred to as the CTD and NTD. The CTD recognizes and binds UDP-sugars via highly conserved PSPG motifs, whereas the NTD loosely binds glycosidic ligands, enabling potential substrate structural diversity [52]. A small mutant library was constructed using the FRISM method based on the hotspots identified in alanine scanning mutagenesis, leading to the discovery of the *UGT85A1*^F217V^ mutant. The volume of the amino acid side chain is reduced in the mutant compared with the *UGT85A1*, and the phenyl ring structure is lost. This not only increases the volume of the substrate pocket but also reduces the π-π stacking interactions with the substrate. MD simulations further explain the mechanism behind the enhanced catalytic efficiency, which provides insights for the study of other glycosyltransferases.

In the fed-batch fermentation process, glycerol functioned as the primary carbon source during the initial growth phase, effectively supporting biomass accumulation and providing sufficient metabolic capacity for subsequent product synthesis. Glucose primarily functioned in UDPG synthesis and was utilized as a secondary carbon source only after glycerol depletion. However, due to the low glucose utilization efficiency of the PTS-deficient strain, relying solely on glucose could limit both cell growth and production capacity. We optimized the feeding strategy to maintain glycerol and glucose concentrations below 10 g/L, thereby achieving a balanced metabolic state and alleviating bottlenecks associated with glucose utilization. The continuous supply of glycerol sustained central carbon metabolism and hydroxytyrosol synthesis, while the controlled glucose concentration minimized inefficient utilization and redirected more carbon flux toward hydroxysalidroside biosynthesis. This dual-carbon source strategy effectively regulated the metabolic network, reduced carbon loss to by-products, and improved target product titer. Future studies could further validate this mechanism through metabolomic and transcriptomic analyses.

In summary, the *de novo* synthesis of hydroxysalidroside was achieved for the first time in microorganisms through UGT screening, construction of synthetic pathways, and strategies for improving glycosylation efficiency, using simple carbon sources. Additionally, the mutant *UGT85A1*^F217V^, which demonstrated enhanced catalytic efficiency, can be further applied to the synthesis of compounds such as salidroside. Based on this study, further modifications to the PTSG region of *UGT85A1* could enhance catalytic activity by promoting UDPG binding within the sugar donor pocket. In addition, obtaining its crystal structure could allow for detailed analysis of its catalytic mechanism. This study also provides valuable insights into the development of *de novo* synthesis pathways for downstream phenylethanoid glycosides.

## CRediT authorship contribution statement

**Xinru Wang:** Writing – review & editing, Writing – original draft, Visualization, Investigation. **Lian Wang:** Writing – review & editing, Investigation. **Qihang Chen:** Writing – review & editing. **Ke Wang:** Writing – review & editing. **Huijing Wang:** Investigation. **Dong Li:** Writing – review & editing. **Song Gao:** Supervision, Funding acquisition. **Weizhu Zeng:** Writing – review & editing. **Jingwen Zhou:** Writing – review & editing, Supervision, Funding acquisition.

## Declaration of competing interest

The authors declare that they have no known competing financial interests or personal relationships that could have appeared to influence the work reported in this paper.
