# Supplementary material for: Efficient production of hydroxysalidroside in Escherichia coli via enhanced glycosylation and semi-rational design of UGT85A1
Source: Synth Syst Biotechnol. 2025 Mar 6;10(2):638–49. doi: 10.1016/j.synbio.2025.03.002 (PMC11957517; doi:10.1016/j.synbio.2025.03.002)
Supplement: Multimedia component 1 [file mmc1.docx]

**Supplementary information**

**Efficient production of hydroxysalidroside in *Escherichia coli* via enhanced glycosylation and semi-rational design of *UGT85A1***

Xinru Wang^1,2^, Lian Wang^1,2^, Qihang Chen^1,2^, Ke Wang^1,2^, Huijing Wang^1,2^, Dong Li^1,2^, Song Gao^1,2^, Weizhu Zeng^1,2^, Jingwen Zhou^1,2,3,4,*^.

^1^ Engineering Research Center of Ministry of Education on Food Synthetic Biotechnology, Jiangnan University, 1800 Lihu Road, Wuxi, Jiangsu 214122, China;

^2^ Science Center for Future Foods, Jiangnan University, 1800 Lihu Road, Wuxi, Jiangsu 214122, China;

^3^ Key Laboratory of Industrial Biotechnology, Ministry of Education and School of Biotechnology, Jiangnan University, 1800 Lihu Road, Wuxi, Jiangsu 214122, China;

^4^ Jiangsu Province Engineering Research Center of Food Synthetic Biotechnology, Jiangnan University, Wuxi 214122, China.

* Correspondence to:

Jingwen Zhou

Science Center for Future Foods, Jiangnan University, 1800 Lihu Rd, Wuxi, Jiangsu 214122, China.

Phone: +86-510-85914371, Fax: +86-510-85914371

E-mail: zhoujw1982@jiangnan.edu.cn

# Supplementary Tables

**Table S1 Strains used in this study.**

| **Strains** | **Description** | **Sources** |
| --- | --- | --- |
| *E. coli* JM109 | Gene cloning | Invitrogen |
| *E. coli* BL21(DE3) | Parent strain | Invitrogen |
| QH01 | *E. coli* BL21 (DE3) containing pETDuet-*UGT85A1* and pCDFDuet-*pgm*-*galU* | This study |
| QH02 | *E. coli* BL21 (DE3) containing  pETDuet-*UGT33* and pCDFDuet-*pgm*-*galU* | This study |
| QH03 | *E. coli* BL21 (DE3) containing  pETDuet-*UGT13* and pCDFDuet-*pgm*-*galU* | This study |
| QH04 | *E. coli* BL21 (DE3) with the deletion of *tyrR, crr, ptsG* and *pheA* | Lab stock[1, 2] |
| QH05 | QH04 containing pRSFDuet-*pgm*-*galU*-*LAAD*-*ADH6-ARO10* and pETDuet-*aroG*^fbr^-*tyrC*-*UGT85A1*-*HpaBC* | This study |
| QH06 | QH04 containing pRSFDuet-*pgm*-*galU*-*LAAD*-*ADH6-ARO10* and pETDuet-*aroG*^fbr^-*tyrC*-*HpaBC*-*UGT85A1* | This study |
| QH07 | QH04 containing pRSFDuet-*pgm*-*galU*-*LAAD*-*HpaBC-ADH6-ARO10* and pETDuet-*aroG*^fbr^-*tyrC*-*UGT85A1* | This study |
| QH08 | QH04 containing pRSFDuet-*pgm*-*galU*-*LAAD*-*ADH6-ARO10-HpaBC* and pETDuet-*aroG*^fbr^-*tyrC*-*UGT85A1* | This study |
| QH09 | *E. coli* BL21 (DE3) with the deletion of *tyrR*, *crr*, *ptsG*, *pheA* and *ushA* containing pRSFDuet-*pgm*-*galU*-*LAAD*-*ADH6-ARO10-HpaBC* and pETDuet-*aroG*^fbr^-*tyrC*-*UGT85A1* | This study |
| QH10 | *E. coli* BL21 (DE3) with the deletion of *tyrR*, *crr*, *ptsG*, *pheA* and *otsA* containing pRSFDuet-*pgm*-*galU*-*LAAD*-*ADH6-ARO10-HpaBC* and pETDuet-*aroG*^fbr^-*tyrC*-*UGT85A1* | This study |
| QH11 | *E. coli* BL21 (DE3) with the deletion of *tyrR*, *crr*, *ptsG*, *pheA* and *ugd* containing pRSFDuet-*pgm*-*galU*-*LAAD*-*ADH6-ARO10-HpaBC* and pETDuet-*aroG*^fbr^-*tyrC*-*UGT85A1* | This study |
| QH12 | *E. coli* BL21 (DE3) with the deletion of *tyrR*, *crr*, *ptsG*, *pheA* and *pgi* containing pRSFDuet-*pgm*-*galU*-*LAAD*-*ADH6-ARO10-HpaBC* and pETDuet-*aroG*^fbr^-*tyrC*-*UGT85A1* | This study |
| QH13 | *E. coli* BL21 (DE3) with the deletion of *tyrR*, *crr*, *ptsG*, *pheA*, *ushA* and *otsA* containing pRSFDuet-*pgm*-*galU*-*LAAD*-*ADH6-ARO10-HpaBC* and pETDuet-*aroG*^fbr^-*tyrC*-*UGT85A1* | This study |
| QH14 | *E. coli* BL21 (DE3) with the deletion of *tyrR*, *crr*, *ptsG*, *pheA*, *ushA* and *ugd* containing pRSFDuet-*pgm*-*galU*-*LAAD*-*ADH6-ARO10-HpaBC* and pETDuet-*aroG*^fbr^-*tyrC*-*UGT85A1* | This study |
| QH15 | *E. coli* BL21 (DE3) with the deletion of *tyrR*, *crr*, *ptsG*, *pheA*, *ushA* and *pgi* containing pRSFDuet-*pgm*-*galU*-*LAAD*-*ADH6-ARO10-HpaBC* and pETDuet-*aroG*^fbr^-*tyrC*-*UGT85A1* | This study |
| QH16 | *E. coli* BL21 (DE3) with the deletion of *tyrR*, *crr*, *ptsG*, *pheA*, *ushA* and *otsA* containing pRSFDuet-*pgm*-*galU*-*LAAD*-*ADH6-ARO10-HpaBC* and pETDuet-*aroG*^fbr^-*tyrC*-*UGT85A1*^F217V^ | This study |

**Table S2 Plasmids used in this study.**

| **Plasmids** | **Description** | **Sources** |
| --- | --- | --- |
| pETDuet-1 | pBR322 with PT7; AmpR | Novagen |
| pCDFDuet-1 | CDF ori with PT7; SmR | Novagen |
| pRSFDuet-1 | RSF ori with PT7; KanR | Novagen |
| pET28a-1 | fi ori with PT7; KanR | Novagen |
| pTarget |  | Lab stock |
| pCas9 | repA101(Ts) kan Pcas-cas9 ParaB-Red lacI Ptrc-sgRNA-pMB1 | Lab stock |
| pCDFDuet-*pgm*-*galU* | pCDFDuet-1 with *pgm* in MCS1 and *galU* in MCS2 | This study |
| pETDuet-*UGT85A1* | pETDuet-1 with *UGT85A1* in MCS1 | This study |
| pETDuet-*UGT33* | pETDuet-1 with *UGT33* in MCS1 | This study |
| pETDuet-*UGT13* | pETDuet-1 with *UGT13* in MCS1 | This study |
| pRSFDuet-*pgm*-*galU*-*LAAD*-*ADH6-ARO10* | pRSFDuet-1 with *pgm*-*galU*-*LAAD* in MCS1 and *ADH6-ARO10* in MCS2 | This study |
| pETDuet-*aroG*^fbr^-*tyrC* | pETDuet-1 with *aroG*^fbr^ and *tyrC* in MCS1 | Lab stock[3] |
| pETDuet-*aroG*^fbr^-*tyrC*-*UGT85A1*-*HpaBC* | pETDuet-1 with *aroG*^fbr-^*tyrC* in MCS1 and *UGT85A1*-*HpaBC* in MCS2 | This study |
| pETDuet-*aroG*^fbr^-*tyrC*-*HpaBC*-*UGT85A1* | pETDuet-1 with *aroG*^fbr-^*tyrC* in MCS1 and *HpaBC*-*UGT85A1* in MCS2 | This study |
| pRSFDuet-*pgm*-*galU*-*LAAD*-*HpaBC-ADH6-ARO10* | pRSFDuet-1 with *pgm*-*galU*-*LAAD* in MCS1 and *HpaBC-ADH6-ARO10* in MCS2 | This study |
| pETDuet-*aroG*^fbr^-*tyrC*-*UGT85A1* | pETDuet-1 with *aroG*^fbr-^*tyrC* in MCS1 and *UGT85A1* in MCS2 | This study |
| pRSFDuet-*pgm*-*galU*-*LAAD*-*ADH6-ARO10-HpaBC* | pRSFDuet-1 with *pgm*-*galU*-*LAAD* in MCS1 and *ADH6-ARO10-HpaBC* in MCS2 | This study |
| pTatget-*ushA* | pTarget with *ushA* in MCS1 | This study |
| pTatget-*otsA* | pTarget with *otsA* in MCS1 | This study |
| pTatget-*ugd* | pTarget with *ugd* in MCS1 | This study |
| pTatget-*pgi* | pTarget with *pgi* in MCS1 | This study |
| pETDuet-*aroG*^fbr^-*tyrC*-*UGT85A1*^H24A^ | pETDuet-1 with *aroG*^fbr-^*tyrC* in MCS1 and *UGT85A1*^H24A^in MCS2 | This study |
| pETDuet-*aroG*^fbr^-*tyrC*-*UGT85A1*^I89A^ | pETDuet-1 with *aroG*^fbr-^*tyrC* in MCS1 and *UGT85A1*^I89A^ in MCS2 | This study |
| pETDuet-*aroG*^fbr^-*tyrC*-*UGT85A1*^C93A^ | pETDuet-1 with *aroG*^fbr-^*tyrC* in MCS1 and *UGT85A1*^C93A^ in MCS2 | This study |
| pETDuet-*aroG*^fbr^-*tyrC*-*UGT85A1*^D126A^ | pETDuet-1 with *aroG*^fbr-^*tyrC* in MCS1 and *UGT85A1*^D126A^ in MCS2 | This study |
| pETDuet-*aroG*^fbr^-*tyrC*-*UGT85A1*^C128A^ | pETDuet-1 with *aroG*^fbr-^*tyrC* in MCS1 and *UGT85A1*^C128A^ in MCS2 | This study |
| pETDuet-*aroG*^fbr^-*tyrC*-*UGT85A1*^W147A^ | pETDuet-1 with *aroG*^fbr-^*tyrC* in MCS1 and *UGT85A1*^W147A^ in MCS2 | This study |
| pETDuet-*aroG*^fbr^-*tyrC*-*UGT85A1*^T148A^ | pETDuet-1 with *aroG*^fbr-^*tyrC* in MCS1 and *UGT85A1*^T148A^ in MCS2 | This study |
| pETDuet-*aroG*^fbr^-*tyrC*-*UGT85A1*^T149A^ | pETDuet-1 with *aroG*^fbr-^*tyrC* in MCS1 and *UGT85A1*^T149A^ in MCS2 | This study |
| pETDuet-*aroG*^fbr^-*tyrC*-*UGT85A1*^F154A^ | pETDuet-1 with *aroG*^fbr-^*tyrC* in MCS1 and *UGT85A1*^F154A^ in MCS2 | This study |
| pETDuet-*aroG*^fbr^-*tyrC*-*UGT85A1*^Y157A^ | pETDuet-1 with *aroG*^fbr-^*tyrC* in MCS1 and *UGT85A1*^Y157A^ in MCS2 | This study |
| pETDuet-*aroG*^fbr^-*tyrC*-*UGT85A1*^P202A^ | pETDuet-1 with *aroG*^fbr-^*tyrC* in MCS1 and *UGT85A1*^P202A^ in MCS2 | This study |
| pETDuet-*aroG*^fbr^-*tyrC*-*UGT85A1*^F204A^ | pETDuet-1 with *aroG*^fbr-^*tyrC* in MCS1 and *UGT85A1*^F204A^ in MCS2 | This study |
| pETDuet-*aroG*^fbr^-*tyrC*-*UGT85A1*^M214A^ | pETDuet-1 with *aroG*^fbr-^*tyrC* in MCS1 and *UGT85A1*^M214A^ in MCS2 | This study |
| pETDuet-*aroG*^fbr^-*tyrC*-*UGT85A1*^F217A^ | pETDuet-1 with *aroG*^fbr-^*tyrC* in MCS1 and *UGT85A1*^F217A^ in MCS2 | This study |
| pETDuet-*aroG*^fbr^-*tyrC*-*UGT85A1*^L219A^ | pETDuet-1 with *aroG*^fbr-^*tyrC* in MCS1 and *UGT85A1*^L219A^ in MCS2 | This study |
| pETDuet-*aroG*^fbr^-*tyrC*-*UGT85A1*^E221A^ | pETDuet-1 with *aroG*^fbr-^*tyrC* in MCS1 and *UGT85A1*^E221A^ in MCS2 | This study |
| pETDuet-*aroG*^fbr^-*tyrC*-*UGT85A1*^T222A^ | pETDuet-1 with *aroG*^fbr-^*tyrC* in MCS1 and *UGT85A1*^T222A^ in MCS2 | This study |
| pETDuet-*aroG*^fbr^-*tyrC*- *UGT85A1*^F403A^ | pETDuet-1 with *aroG*^fbr-^*tyrC* in MCS1 and *UGT85A1*^F403A^ in MCS2 | This study |
| pETDuet-*aroG*^fbr^-*tyrC*- *UGT85A1*^D405A^ | pETDuet-1 with *aroG*^fbr-^*tyrC* in MCS1 and *UGT85A1*^D405A^ in MCS2 | This study |
| pETDuet-*aroG*^fbr^-*tyrC*-*UGT85A1*^F217L^ | pETDuet-1 with *aroG*^fbr-^*tyrC* in MCS1 and *UGT85A1*^F217L^ in MCS2 | This study |
| pETDuet-*aroG*^fbr^-*tyrC*-*UGT85A1*^F217G^ | pETDuet-1 with *aroG*^fbr-^*tyrC* in MCS1 and *UGT85A1*^F217G^ in MCS2 | This study |
| pETDuet-*aroG*^fbr^-*tyrC*-*UGT85A1*^F217V^ | pETDuet-1 with *aroG*^fbr-^*tyrC* in MCS1 and *UGT85A1*^F217V^ in MCS2 | This study |
| pETDuet-*aroG*^fbr^-*tyrC*-*UGT85A1*^F217A/T149S^ | pETDuet-1 with *aroG*^fbr-^*tyrC* in MCS1 and *UGT85A1*^F217A/T149S^ in MCS2 | This study |
| pETDuet-*aroG*^fbr^-*tyrC*- *UGT85A1*^F217A/T149D^ | pETDuet-1 with *aroG*^fbr-^*tyrC* in MCS1 and *UGT85A1*^F217A/T149D^ in MCS2 | This study |
| pETDuet-*aroG*^fbr^-*tyrC*- *UGT85A1*^F217A/T149E^ | pETDuet-1 with *aroG*^fbr-^*tyrC* in MCS1 and *UGT85A1*^F217A/T149E^ in MCS2 | This study |
| pETDuet-*aroG*^fbr^-*tyrC*- *UGT85A1*^F217A/F204Y^ | pETDuet-1 with *aroG*^fbr-^*tyrC* in MCS1 and *UGT85A1*^F217A/F204Y^ in MCS2 | This study |
| pETDuet-*aroG*^fbr^-*tyrC*- *UGT85A1*^F217A/F204P^ | pETDuet-1 with *aroG*^fbr-^*tyrC* in MCS1 and *UGT85A1*^F217A/F204P^ in MCS2 | This study |
| pETDuet-*aroG*^fbr^-*tyrC*- *UGT85A1*^F217A/F204W^ | pETDuet-1 with *aroG*^fbr-^*tyrC* in MCS1 and *UGT85A1*^F217A/F204W^ in MCS2 | This study |
| pETDuet-*aroG*^fbr^-*tyrC*- *UGT85A1*^F217A/A404D^ | pETDuet-1 with *aroG*^fbr-^*tyrC* in MCS1 and *UGT85A1*^F217A/A404D^ in MCS2 | This study |
| pETDuet-*aroG*^fbr^-*tyrC*- *UGT85A1*^F217A/A404E^ | pETDuet-1 with *aroG*^fbr-^*tyrC* in MCS1 and *UGT85A1*^F217A/A404E^ in MCS2 | This study |
| pETDuet-*aroG*^fbr^-*tyrC*- *UGT85A1*^F217A/D405E^ | pETDuet-1 with *aroG*^fbr-^*tyrC* in MCS1 and *UGT85A1*^F217A/D405E^ in MCS2 | This study |
| pET28a-*UGT85A1* | pET28a-1 with *UGT85A1* in MCS | This study |
| pET28a-*UGT85A1*^F217V^ | pET28a-1 with *UGT85A1*^F217V^ in MCS | This study |

**Table S3 Primers used in this study.**

| **Primers** | **Sequence (5'-3')** |
| --- | --- |
| Duet-F | TAATAATGCTTAAGTCGAACAGAAAGTAATCGTATTGTACACGGCCG |
| Duet-R | CATGGTATATCTCCTTCTTAAAGTTAAACAAAATTATTTCTAGAGGG |
| *UGT85A1*-F | ATGGGTAGCCAGATCATCCACA |
| *UGT85A1*-R | CGAACACAAACTGGGTAGTTCCGTTTAA |
| *UGT33*-F | ATGAGCCTGATCGAAAAACCGC |
| *UGT33*-R | TTAACGGATGTGTTTGGTTTTGCTCA |
| *UGT13*-F | ATGGGCAGCCTGGGCGCGGC |
| *UGT13*-R | GTGCTGCTGGCGAAAGAAAGCTAA |
| CDF-M2-F | TTAACCTAGGCTGCTGCCAC |
| CDF-M2-R | CATATGTATATCTCCTTCTTATACTTAACT |
| *HpaBC*-F | ATGAAACCAGAAGATTTCCGCGC |
| *HpaBC*-R | TTAAATCGCAGCTTCCATTTCCAGC |
| *UGT85A1*-F | ATGGGTAGCCAGATCATCCACAAC |
| *UGT85A1*-R | GGGTCAGAAAAGCCAGGACTAA |
| *aroG*^fbr^-F | ATGAATTATCAGAACGACGATTTACG |
| *aroG*^fbr^-R | TTACCCGCGACGCGCTTTTACT |
| *tyrC-*F | TGACCGTTTTCAAACACATCGCG |
| *tyrC-*R | CCGATCACGATATCCACCCGTAA |
| ET-pla-F | AAGCTTGCGGCCGCATAATGCT |
| ET-pla-R | GCCCATGGTATATCTCCTTCTTAAAGTTAAAC |
| *pgm*-F | ATGGCAATCCACAATCGTGCAG |
| *pgm*-R | TTACGCGTTTTTCAGAACTTCGCT |
| *galU*-F | ATGGCTGCCATTAATACGAAAGTC |
| *galU*-R | TTACTTCTTAATGCCCATCTCTTCTTCAAGC |
| *LAAD*-F | AACATCTCTCGTCGTAAACTGC |
| *LAAD*-R | CGTTCAGCCTGGATCGTTTCAAAAAATAA |
| *ARO10*-F | ATGGCACCTGTTACAATTGAAAAGT |
| *ARO10*-R | GAAGCAGCGGCACTTAAAAGAAATAAAAAATAG |
| *ADH6-F* | ATGTCTTATCCTGAGAAATTTGAAGGTATCG |
| *ADH6-R* | CGGCTACGACAAAGAATTTTCAGACTAG |
| *ushA*-F | AGTGGGGCAAATACGTGGGAGTTTTAGAGCTAGAAATAGCAAGTT |
| *ushA*-R | TCCCACGTATTTGCCCCACTACTAGTATTATACCTAGGACTGAGC |
| *ushA*-Up-F | TTGGCAACGACCATGGCATC |
| *ushA*-Up-R | ACTTCTCTCCCTGACCTGATTTCAAC |
| *ushA*-Down-F | ATCAGGTCAGGGAGAGAAGTTCCGAAAGTGCCGGATGTTTG |
| *ushA*-Down-R | AATTACTCGAAAAAAACAAGATTTCGTTTCAAATCC |
| *ostA*-F | TGACGAAGTTGCAGCTGCGCGTTTTAGAGCTAGAAAT |
|  | AGCAAGTT |
| *ostA*-R | GCGCAGCTGCAACTTCGTCAACTAGTATTATACCTAGGACTGAGC |
| *ostA*-Up-F | CGTCGTTTCTCCATTAGGAGTAAAGCTTT |
| *ostA*-Up-R | GAGCGGTTAATCTCCCGTAAGTGGAA |
| *ostA*-Down-F | TTAGATACTACGACTAAACGACTCATAGTCATCA |
| *ostA*-Down-R | AACACATATCGTTCATCTGCCGGATG |
| *ugd*-F | TTGCGGCTTGCGTGACAAAAGTTTTAGAGCTAGAAATAGCAAGTT |
| *ugd*-R | TTTTGTCACGCAAGCCGCAAACTAGTATTATACCTAGGACTGAGC |
| *ugd*-Up-F | GTTTTTCTGGTTCTTCCTGATTATCCAGCG |
| *ugd*-Up-R | CATCCTGTTATCAGGGCTATTTACGCC |
| *ugd*-Down-F | TTCATGCTCTCAGAATTAACTTAACTGTGAATCA |
| *ugd*-Down-R | TAACCTGCTGCTGGCTCCGTA |
| *pgi*-F | TGCTGGCGCTGATTGGCATCGTTTTAGAGCTAGAAATAGCAAGTT |
| *pgi*-R | GATGCCAATCAGCGCCAGCAACTAGTATTATACCTAGGACTGAGC |
| *pgi*-Up-F | TTTTCAGCCTTGGCACAAGGGA |
| *pgi*-Up-R | TAGCAATACTCTTCTGATTTTGAGAATTGTGACT |
| *pgi*-Down-F | TCATCGTCGATATGTAGGCCGGATAAG |
| *pgi*- Down-R | ATAACAATTTCCCTTCATTGAATGAATGGAGATTTAC |
| H24A-F | CAAGGTGCGATTAACCCGATGATG |
| H24A-F | TTAATCGCACCTTGCGCCGGGTACGGAACACAAA |
| I89A-F | TCAGGATGCGACCGCTCTG |
| I89A-R | CGGTCGCATCCTGAGTAGCATCCATATCGGTTTCCGGC |
| C93A-F | CTGGCGGAAAGCACCATGAAA |
| C93A-R | TGCTTTCCGCCAGAGCGGTAATATCCTGAGTAGCATCC |
| D126A-F | TGAGCGCGGGTTGCATGAGCTTCACTCTGGACGTGG |
| D126A-R | GCAACCCGCGCTCACGATACAGCTAACCGGCGGAA |
| C128A-F | TGGTGCGATGAGCTTCACTCTG |
| C128A-R | GAAGCTCATCGCACCATCGCTCACGATACAGCTAACCG |
| W147A-F | GTTTGCGACCACTAGCGGT |
| W147A-R | TAGTGGTCGCAAACAGAACTTCCGGAACACCCAGTTCT |
| T148A-F | TTGGGCGACTAGCGGTTGC |
| T148A-R | CCGCTAGTCGCCCAAAACAGAACTTCCGGAACACCC |
| T149A-F | GCGAGCGGTTGCGCTTTC |
| T149A-R | AGCGCAACCGCTCGCGGTCCAAAACAGAACTTCCGGAAC |
| F154A-F | TTGCGCTGCGCTGGCGTATCTGCACTTTTACCTGTTTATCGAAAA |
| F154A-R | CAGATACGCCAGCGCAGCGCAACCGCTAGTGG |
| Y157A-F | TGGCGGCGCTGCACTTTTACCTGTTTATCGAAAAAGGCCTGTG |
| Y157A-R | GTGCAGCGCCGCCAGGAAAGCGCAACCGCTAGT |
| P202A-F | ATATCGCGTCTTTTATCCGCACGACCAACCC |
| P202A-R | AAAAGACGCGATATCTTTCAGTTTCACGTTTTTCATAGTCGGAATA |
| F204A-F | CATCTGCGATCCGCACGACCAACCCGGACGATG |
| F204A-R | GCGGATCGCAGATGGGATATCTTTCAGTTTCACGTTTTTCATAGTCG |
| M214A-F | GATGTGGCGATCTCTTTCGCGCTGCGTGAAACTGAA |
| M214A-R | GAGATCGCCACATCGTCCGGGTTGGTCGTGC |
| F217A-F | ATCTCTGCGGCGCTGCGTGAAACTGAACGTGCAAA |
| F217A-R | AGCGCCGCAGAGATCATCACATCGTCCGGGTTGGTC |
| L219A-F | TCGCGGCGCGTGAAACTGAACGTGCAAAACGTGCAA |
| L219A-R | TTCACGCGCCGCGAAAGAGATCATCACATCGTCCGGG |
| E221A-F | TGCGTGCGACTGAACGTGCAAAACGTGCAAGCG |
| E221A-R | TTCAGTCGCACGCAGCGCGAAAGAGATCATCACATCG |
| T222A-F | CGTGAAGCGGAACGTGCAAAACGTGCAAGCGCTAT |
| T222A-R | CGTTCCGCTTCACGCAGCGCGAAAGAGATCATCACA |
| F403A-F | CCGTTCGCGGCTGACCAGCAGATGAACTGTAAATTCTGCTG |
| F403A-R | TCAGCCGCGAACGGCCAGCACACCATCGGAACG |
| D405A-F | TCGCTGCGCAGCAGATGAACTGTAAATTCTGCTGCGATGAATG |
| D405A-R | CTGCTGCGCAGCGAAGAACGGCCAGCACACCA |
| F217L-F | ATCTCTCTGGCGCTGCGTGAAACTGAACGTGCAAA |
| F217L-R | AGCGCCAGAGAGATCATCACATCGTCCGGGTTGGTC |
| F217G-F | ATCTCTGGTGCGCTGCGTGAAACTGAACGTGCAAA |
| F217G-R | AGCGCACCAGAGATCATCACATCGTCCGGGTTGGTC |
| F217V-F | ATCTCTGTGGCGCTGCGTGAAACTGAACGTGCAAA |
| F217V-R | AGCGCCACAGAGATCATCACATCGTCCGGGTTGGTC |
| F217A/T149S-F | ACCAGCAGCGGTTGCGC |
| F217A/T149S-R | CAACCGCTGCTGGTCCAAAACAGAA |
| F217A/T149D-F | ACCGACAGCGGTTGCGC |
| F217A/T149D-R | CAACCGCTGTCGGTCCAAAACAGAA |
| F217A/T149E-F | ACCGAAAGCGGTTGCGC |
| F217A/T149E-R | CAACCGCTTTCGGTCCAAAACAGAA |
| F217A/F204Y-F | CCCATCTTACATCCGCACGAC |
| F217A/F204Y-R | GCGGATGTAAGATGGGATATCTTTCA |
| F217A/F204P-F | CCCATCTCCAATCCGCACGAC |
| F217A/F204P-R | GCGGATTGGAGATGGGATATCTTTCA |
| F217A/F204W-F | CCCATCTTGGATCCGCACGAC |
| F217A/F204W-R | GCGGATCCAAGATGGGATATCTTTCA |
| F217A/A404D-F | GTTCTTCGACGACCAGCAGATG |
| F217A/A404D-R | TGGTCGTCGAAGAACGGCCA |
| F217A/A404E-F | GTTCTTCGAAGACCAGCAGATG |
| F217A/A404E-R | TGGTCTTCGAAGAACGGCCA |
| F217A/A405D-F | TCGCTGAACAGCAGATGAACTGTAAATT |
| F217A/A405D-R | CTGCTGTTCAGCGAAGAACGG |
| pET28a-F | AAAGCCAGGACTAAGCGACCCATTTGCTGTCCAC |
| pET28a-R | ATCTGGCTACCCATCACCACCACCACCACCACTG |

**Table S4 Nucleotide sequences of codon-optimized genes in this study**

| **Gene** | **Sequence (5'-3')** |
| --- | --- |
| *UGT85A1* | ATGGGTAGCCAGATCATCCACAACAGCCAGAAACCGCACGTTGTTTGTGTTCCGTACCCGGCGCAAGGTCACATTAACCCGATGATGCGTGTTGCAAAACTGCTGCACGCGCGTGGCTTCTACGTGACCTTCGTTAACACCGTTTATAACCACAACCGTTTCCTGCGCTCTCGTGGTTCCAACGCACTGGATGGTCTGCCTTCTTTCCGTTTCGAATCTATCGCTGATGGTCTGCCGGAAACCGATATGGATGCTACTCAGGATATTACCGCTCTGTGCGAAAGCACCATGAAAAACTGCTTAGCACCGTTCCGTGAACTGCTGCAGCGTATTAACGCGGGTGACAACGTTCCGCCGGTTAGCTGTATCGTGAGCGATGGTTGCATGAGCTTCACTCTGGACGTGGCTGAAGAACTGGGTGTTCCGGAAGTTCTGTTTTGGACCACTAGCGGTTGCGCTTTCCTGGCGTATCTGCACTTTTACCTGTTTATCGAAAAAGGCCTGTGTCCGCTGAAAGATGAATCTTACCTGACTAAAGAATATCTGGAAGATACTGTGATCGACTTTATTCCGACTATGAAAAACGTGAAACTGAAAGATATCCCATCTTTTATCCGCACGACCAACCCGGACGATGTGATGATCTCTTTCGCGCTGCGTGAAACTGAACGTGCAAAACGTGCAAGCGCTATCATTCTGAACACCTTCGATGACCTGGAACATGATGTTGTTCATGCAATGCAGAGC |
|  | ATCCTGCCGCCGGTGTACTCTGTGGGTCCGCTGCACCTGCTGGCTAACCGTGAAATTGAAGAAGGTTCTGAAATCGGTATGATGAGCTCCAACCTGTGGAAAGAAGAAATGGAATGCCTGGATTGGCTGGATACCAAAACCCAGAACAGCGTTATTTACATCAACTTCGGCTCTATCACCGTTCTGTCCGTGAAACAGCTGGTTGAATTTGCGTGGGGCCTGGCGGGCTCTGGTAAAGAATTCCTGTGGGTGATCCGTCCGGATCTGGTTGCTGGTGAAGAAGCGATGGTTCCGCCGGATTTCTTGATGGAAACCAAAGATCGTTCTATGCTGGCGAGCTGGTGCCCGCAAGAAAAAGTTCTGTCCCATCCGGCAATCGGTGGCTTCCTGACCCATTGCGGCTGGAACAGCATTCTGGAAAGCCTGTCTTGCGGCGTTCCGATGGTGTGCTGGCCGTTCTTCGCTGACCAGCAGATGAACTGTAAATTCTGCTGCGATGAATGGGACGTGGGTATCGAAATCGGCGGTGATGTTAAACGTGAAGAAGTTGAAGCGGTAGTGCGTGAACTGATGGATGGTGAAAAAGGTAAAAAGATGCGTGAAAAAGCAGTTGAATGGCAGCGTCTGGCTGAAAAAGCTACCGAACACAAACTGGGTAGTTCCGTTATGAACTTCGAAACCGTTGTGTCTAAATTCCTGCTGGGTCAGAAAAGCCAGGACTAA |
| *UGT85A1*^F217A^ | ATGGGTAGCCAGATCATCCACAACAGCCAGAAACCGCACGTTGTTTGTGTTCCGTACCCGGCGCAAGGTCACATT |
|  | AACCCGATGATGCGTGTTGCAAAACTGCTGCACGCGCGTGGCTTCTACGTGACCTTCGTTAACACCGTTTATAACCACAACCGTTTCCTGCGCTCTCGTGGTTCCAACGCACTGGATGGTCTGCCTTCTTTCCGTTTCGAATCTATCGCTGATGGTCTGCCGGAAACCGATATGGATGCTACTCAGGATATTACCGCTCTGTGCGAAAGCACCATGAAAAACTGCTTAGCACCGTTCCGTGAACTGCTGCAGCGTATTAACGCGGGTGACAACGTTCCGCCGGTTAGCTGTATCGTGAGCGATGGTTGCATGAGCTTCACTCTGGACGTGGCTGAAGAACTGGGTGTTCCGGAAGTTCTGTTTTGGACCACTAGCGGTTGCGCTTTCCTGGCGTATCTGCACTTTTACCTGTTTATCGAAAAAGGCCTGTGTCCGCTGAAAGATGAATCTTACCTGACTAAAGAATATCTGGAAGATACTGTGATCGACTTTATTCCGACTATGAAAAACGTGAAACTGAAAGATATCCCATCTTTTATCCGCACGACCAACCCGGACGATGTGATGATCTCTGCGGCGCTGCGTGAAACTGAACGTGCAAAACGTGCAAGCGCTATCATTCTGAACACCTTCGATGACCTGGAACATGATGTTGTTCATGCAATGCAGAGCATCCTGCCGCCGGTGTACTCTGTGGGTCCGCTGCACCTGCTGGCTAACCGTGAAATTGAAGAAGGTTCTGAAATCGGTATGATGAGCTCCAACCTGTGGAAAGAAGAAATGGAATGCCTGGATTGGCTGGATACCAAAACCCAGAACAG |
|  | CGTTATTTACATCAACTTCGGCTCTATCACCGTTCTGTCCGTGAAACAGCTGGTTGAATTTGCGTGGGGCCTGGCGGGCTCTGGTAAAGAATTCCTGTGGGTGATCCGTCCGGATCTGGTTGCTGGTGAAGAAGCGATGGTTCCGCCGGATTTCTTGATGGAAACCAAAGATCGTTCTATGCTGGCGAGCTGGTGCCCGCAAGAAAAAGTTCTGTCCCATCCGGCAATCGGTGGCTTCCTGACCCATTGCGGCTGGAACAGCATTCTGGAAAGCCTGTCTTGCGGCGTTCCGATGGTGTGCTGGCCGTTCTTCGCTGACCAGCAGATGAACTGTAAATTCTGCTGCGATGAATGGGACGTGGGTATCGAAATCGGCGGTGATGTTAAACGTGAAGAAGTTGAAGCGGTAGTGCGTGAACTGATGGATGGTGAAAAAGGTAAAAAGATGCGTGAAAAAGCAGTTGAATGGCAGCGTCTGGCTGAAAAAGCTACCGAACACAAACTGGGTAGTTCCGTTATGAACTTCGAAACCGTTGTGTCTAAATTCCTGCTGGGTCAGAAAAGCCAGGACTAA |
| *UGT85A1*^F217V^ | ATGGGTAGCCAGATCATCCACAACAGCCAGAAACCGCACGTTGTTTGTGTTCCGTACCCGGCGCAAGGTCACATTAACCCGATGATGCGTGTTGCAAAACTGCTGCACGCGCGTGGCTTCTACGTGACCTTCGTTAACACCGTTTATAACCACAACCGTTTCCTGCGCTCTCGTGGTTCCAACGCACTGGATGGTCTGCCTTCTTTCCGTTTCGAATCTATCGCT |
|  | GATGGTCTGCCGGAAACCGATATGGATGCTACTCAGGATATTACCGCTCTGTGCGAAAGCACCATGAAAAACTGCTTAGCACCGTTCCGTGAACTGCTGCAGCGTATTAACGCGGGTGACAACGTTCCGCCGGTTAGCTGTATCGTGAGCGATGGTTGCATGAGCTTCACTCTGGACGTGGCTGAAGAACTGGGTGTTCCGGAAGTTCTGTTTTGGACCACTAGCGGTTGCGCTTTCCTGGCGTATCTGCACTTTTACCTGTTTATCGAAAAAGGCCTGTGTCCGCTGAAAGATGAATCTTACCTGACTAAAGAATATCTGGAAGATACTGTGATCGACTTTATTCCGACTATGAAAAACGTGAAACTGAAAGATATCCCATCTTTTATCCGCACGACCAACCCGGACGATGTGATGATCTCTGTGGCGCTGCGTGAAACTGAACGTGCAAAACGTGCAAGCGCTATCATTCTGAACACCTTCGATGACCTGGAACATGATGTTGTTCATGCAATGCAGAGCATCCTGCCGCCGGTGTACTCTGTGGGTCCGCTGCACCTGCTGGCTAACCGTGAAATTGAAGAAGGTTCTGAAATCGGTATGATGAGCTCCAACCTGTGGAAAGAAGAAATGGAATGCCTGGATTGGCTGGATACCAAAACCCAGAACAGCGTTATTTACATCAACTTCGGCTCTATCACCGTTCTGTCCGTGAAACAGCTGGTTGAATTTGCGTGGGGCCTGGCGGGCTCTGGTAAAGAATTCCTGTGGGTGATCCGTCCGGATCTGGTTGCTGGTGAAGAAGCGATGGTTCCGCCGGATTTCTTGATGGAAACCAAAGATCGTTCTATGCTGGCGAGCTGGTGCCCGCAAGAAAAAGTTCTGTCCCATCCGGCAATCGGTGGCTTCCTGACCCATTGCGGCTGGAACAGCATTCTGGAAAGCCTGTCTTGCGGCGTTCCGATGGTGTGCTGGCCGTTCTTCGCTGACCAGCAGATGAACTGTAAATTCTGCTGCGATGAATGGGACGTGGGTATCGAAATCGGCGGTGATGTTAAACGTGAAGAAGTTGAAGCGGTAGTGCGTGAACTGATGGATGGTGAAAAAGGTAAAAAGATGCGTGAAAAAGCAGTTGAATGGCAGCGTCTGGCTGAAAAAGCTACCGAACACAAACTGGGTAGTTCCGTTATGAACTTCGAAACCGTTGTGTCTAAATTCCTGCTGGGTCAGAAAAGCCAGGACTAA |
| *aroG^fbr^* | ATGAATTATCAGAACGACGATTTACGCATCAAAGAAATCAAAGAGTTACTTCCTCCTGTCGCATTGCTGGAAAAATTCCCCGCTACTGAAAATGCCGCGAATACGGTTGCCCATGCCCGAAAAGCGATCCATAAGATCCTGAAAGGTAATGATGATCGCCTGTTGGTTGTGATTGGCCCATGCTCAATTCATGATCCTGTCGCGGCAAAAGAGTATGCCACTCGCTTGCTGGCGCTGCGTGAAGAGCTGAAAGATGAGCTGGAAATCGTAATGCGCGTCTATTTTGAAAAGCCGCGTACCACGGTGGGCTGGAAAGGGCTGATTAACGATCCGCATATGGATAATAGCTTCCAGATCAACGACGGTCTGCGTAT |
|  | AGCCCGTAAATTGCTGCTTGATATTAACGACAGCGGTCTGCCAGCGGCAGGTGAGTTTCTCGATATGATCACCCCACAATATCTCGCTGACCTGATGAGCTGGGGCGCAATTGGCGCACGTACCACCGAATCGCAGGTGCACCGCGAACTGGCATCAGGGCTTTCTTGTCCGGTCGGCTTCAAAAATGGCACCGACGGTACGATTAAAGTGGCTATCGATGCCATTAATGCCGCCGGTGCGCCGCACTGCTTCCTGTCCGTAACGAAATGGGGGCATTCGGCGATTGTGAATACCAGCGGTAACGGCGATTGCCATATCATTCTGCGCGGCGGTAAAGAGCCTAACTACAGCGCGAAGCACGTTGCTGAAGTGAAAGAAGGGCTGAACAAAGCAGGCCTGCCAGCACAGGTGATGATCGATTTCAGCCATGCTAACTCGTCCAAACAATTCAAAAAGCAGATGGATGTTTGTGCTGACGTTTGCCAGCAGATTGCCGGTGGCGAAAAGGCCATTATTGGCGTGATGGTGGAAAGCCATCTGGTGGAAGGCAATCAGAGCCTCGAGAGCGGGGAGCCGCTGGCCTACGGTAAGAGCATCACCGATGCCTGCATCGGCTGGGAAGATACCGATGCTCTGTTACGTCAACTGGCGAATGCAGTAAAAGCGCGTCGCGGGTAA |

**Table S5 Kinetic Parameters of *UGT85A1* and *UGT85A1*^F217V^ toward hydroxytyrosol**

| **Enzyme** | ***K*_m_ (mM)** | ***k*_cat_ (s^-1^)** | ***k*_cat_/*K*_m_ (mM^-1^ s^-1^)** |
| --- | --- | --- | --- |
| ***UGT85A1*** | 0.55 ± 0.05 | 1.45 ± 0.02 | 2.64 ± 0.24 |
| ***UGT85A1*^F217V^** | 0.47 ± 0.04 | 2.52 ± 0.07 | 5.36 ± 0.28 |

#
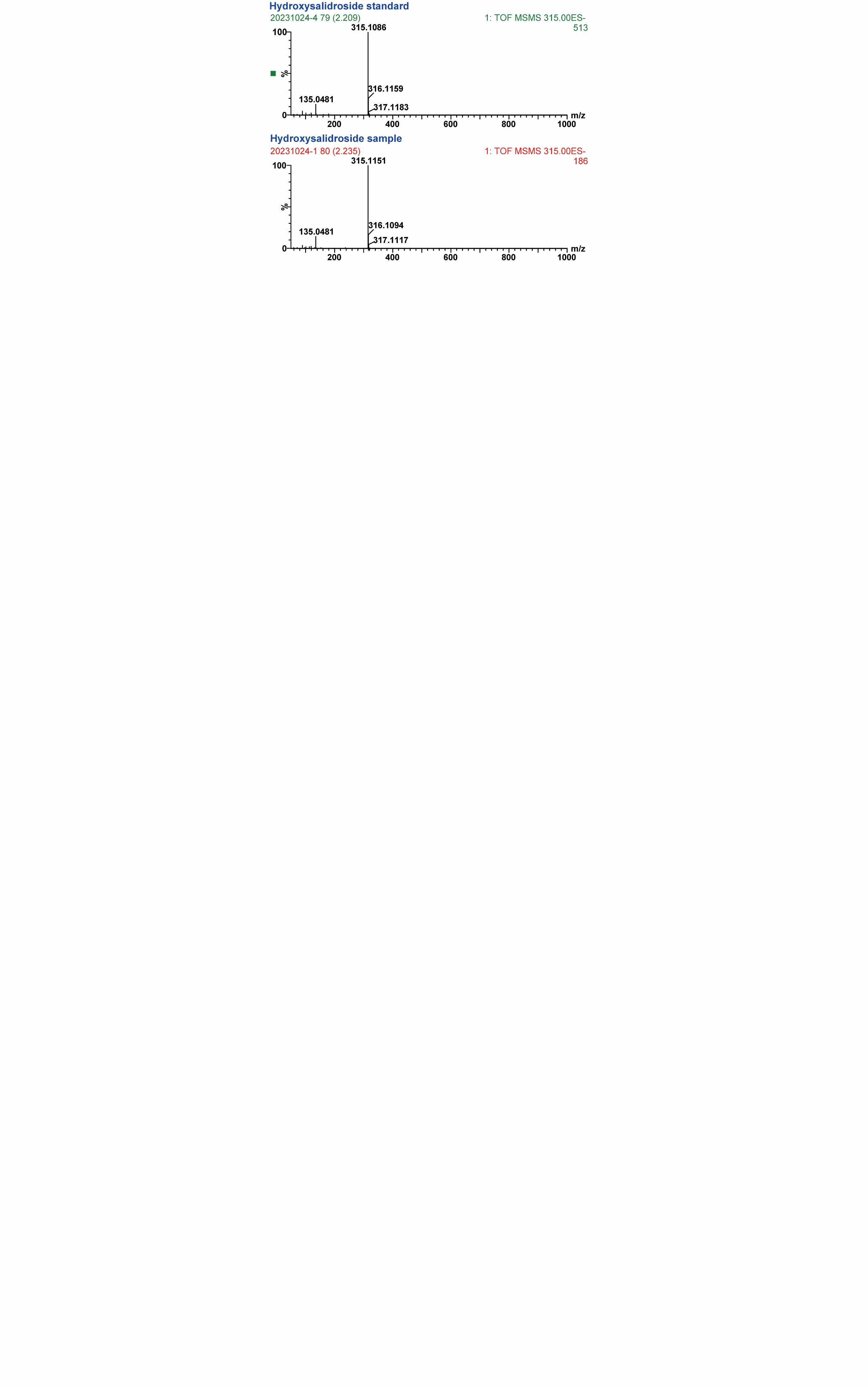
Supplementary Figures

**Figure S1 Identification of hydroxysalidroside by LC−MS**

**
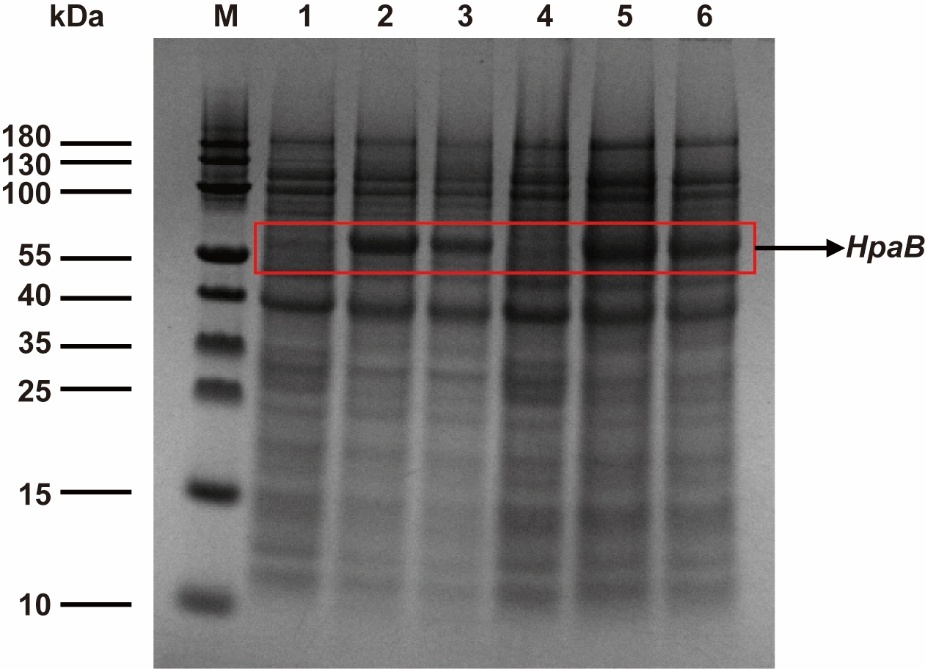
Figure S2. SDS-PAGE analysis of *HpaB*.**

Expression of *HpaB* in *E. coli* BL21(DE3) Δ*tyrR*Δ*ptsG*Δ*crr*Δ*pheA*, the loading volume for each well was 10 µL. M, protein marker; lanes 1, crude enzyme from *E. coli* harboring the empty pETDuet-1 vector; lanes 2, crude enzyme from *E. coli* harboring the pETDuet-1 from QH05; lanes 3, crude enzyme from *E. coli* harboring the pETDuet-1 from QH06; lanes 4, crude enzyme from *E. coli* harboring the empty pRSFDuet-1 vector; lanes 5, crude enzyme from *E. coli* harboring the pRSFDuet-1 from QH07; lanes 6, crude enzyme from *E. coli* harboring the pRSFDuet-1 from QH08.

**
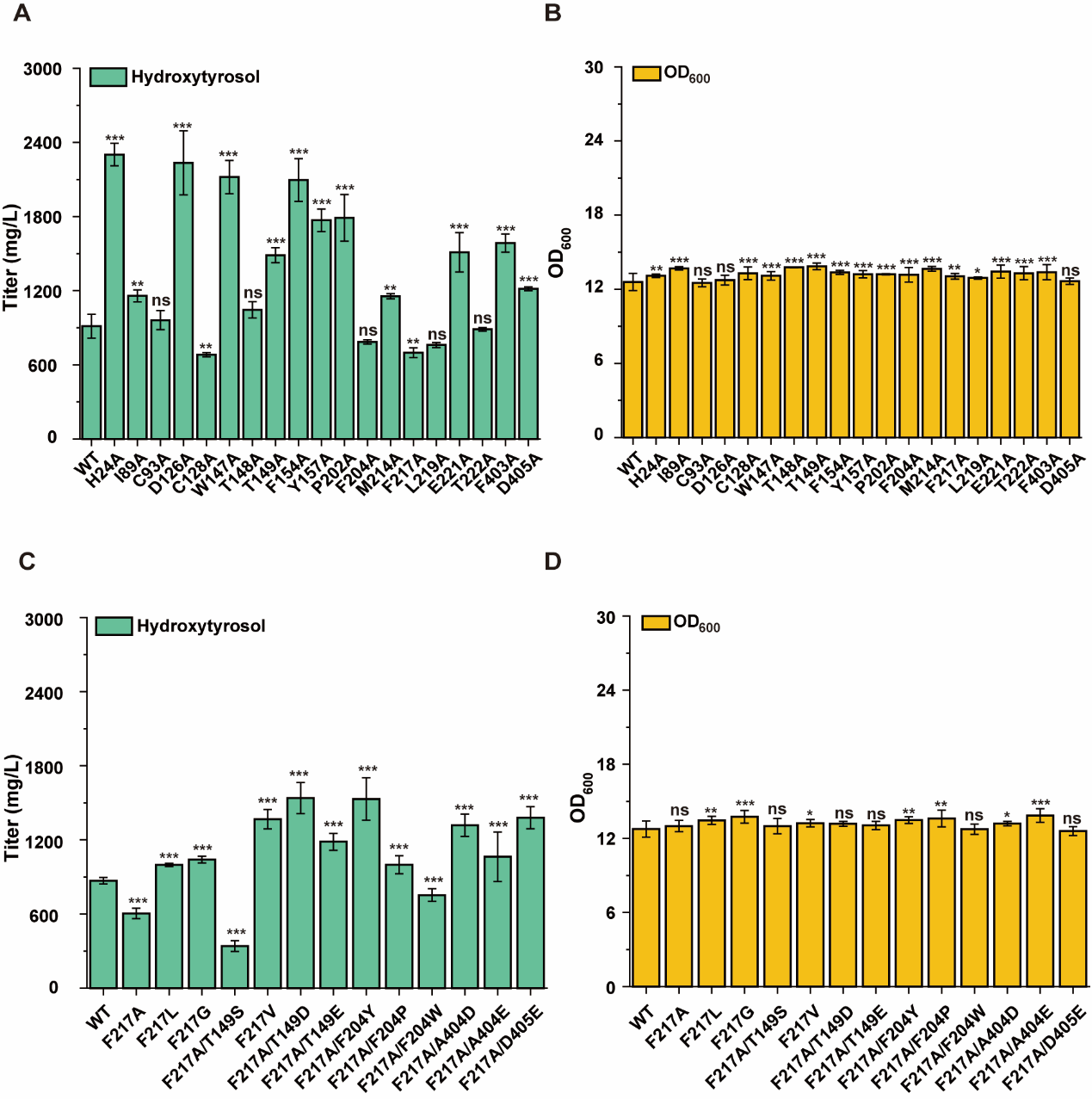
**

**Figure S3 Supporting data on hydroxytyrosol titer and OD₆₀₀ in shake-flask fermentation of strains with mutants.**

(A) Hydroxytyrosol titer of strains harboring alanine mutants. (B) OD_600_ of strains harboring alanine mutants. (C) Hydroxytyrosol titer of strains harboring semi-rational design mutants. (D) OD_600_ of strains harboring semi-rational design mutants. Data shown are mean ± SD of three independent experiments. Statistical significance was determined using one-way ANOVA followed by Tukey's post-hoc test. Asterisks indicate significant differences (* p ≤ 0.05, ** p ≤ 0.01, *** p ≤ 0.001).

**
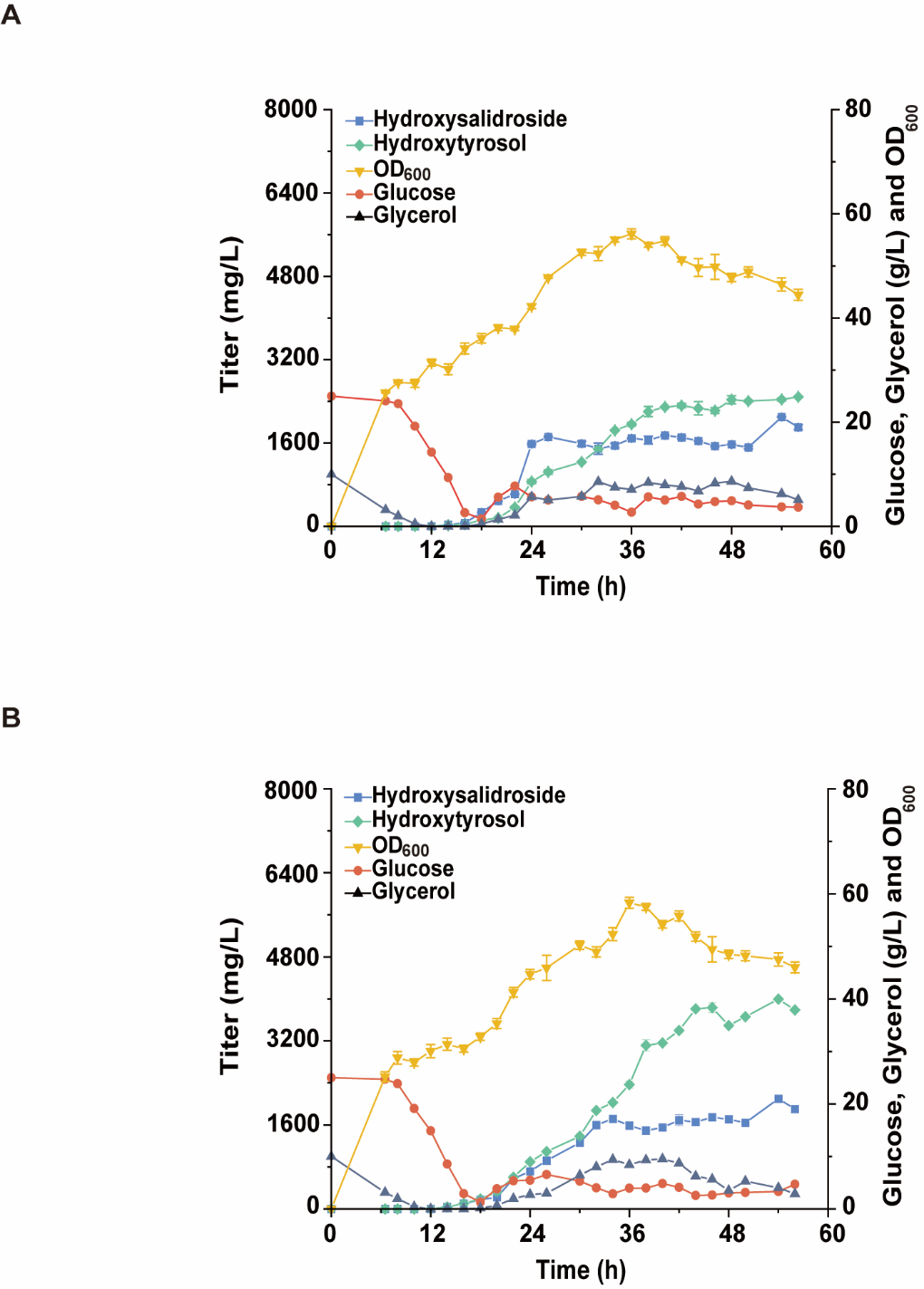
**

**Figure S4 *De novo* biosynthesis of hydroxysalidroside in a 5 L fermenter before optimizing the feeding strategy.**

(A) Biosynthesis of hydroxysalidroside by strain QH13. (B) Biosynthesis of hydroxysalidroside by strain QH16. Data shown are mean ± SD of two independent experiments.

# Reference

[1] Wang L; Wang H; Chen J; Hu M; Shan X; Zhou J, Efficient Production of Chlorogenic Acid in Escherichia coli Via Modular Pathway and Cofactor Engineering. J Agric Food Chem 2023**;**71(41):15204-12. <https://doi.org/10.1021/acs.jafc.3c04419>.

[2] Wang L; Wang H; Chen J; Qin Z; Yu S; Zhou J, Coordinating caffeic acid and salvianic acid A pathways for efficient production of rosmarinic acid in Escherichia coli. Metab Eng 2023**;**76:29-38. <https://doi.org/10.1016/j.ymben.2023.01.002>.

[3] Wang L; Li N; Yu S; Zhou J, Enhancing caffeic acid production in Escherichia coli by engineering the biosynthesis pathway and transporter. Bioresour Technol 2023**;**368:128320. <https://doi.org/10.1016/j.biortech.2022.128320>.
